# Expression of Neuronal Nicotinic Acetylcholine Receptor and Early Oxidative DNA Damage in Aging Rat Brain—The Effects of Memantine

**DOI:** 10.3390/ijms26041634

**Published:** 2025-02-14

**Authors:** Małgorzata Anna Lewandowska, Agata Różycka, Teresa Grzelak, Bartosz Kempisty, Paweł Piotr Jagodziński, Margarita Lianeri, Jolanta Dorszewska

**Affiliations:** 1 Faculty of Medicine, Poznan Medical University, 55 Bulgarska St., 60-320 Poznan, Poland; mal2015lewandowska@gmail.com; 2 Department of Biochemistry and Molecular Biology, Poznan University of Medical Sciences, 6 Świecickiego St., 60-781 Poznan, Poland; pjagodzi@ump.edu.pl (P.P.J.); margarita.lianeri@gmail.com (M.L.); 3 Department of Physiology, Poznan University of Medical Sciences, 6 Świecickiego St., 60-781 Poznan, Poland; 4 Department of Human Morphology and Embryology, Division of Anatomy, Wrocław Medical University, 50-368 Wroclaw, Poland; kempistybartosz@gmail.com; 5 Institute of Veterinary Medicine, Nicolaus Copernicus University, 87-100 Torun, Poland; 6 Physiology Graduate Faculty, North Carolina State University, Raleigh, NC 27695, USA; 7 Center of Assisted Reproduction, Department of Obstetrics and Gynecology, University Hospital and Masaryk University, 625 00 Brno, Czech Republic; 8 Laboratory of Neurobiology, Department of Neurology, Poznan University of Medical Sciences, 49 Przybyszewskiego St., 60-355 Poznan, Poland; jolanta.dorszewska@ump.edu.pl

**Keywords:** brain, aging, nicotinic receptor, oxidative stress, memantine

## Abstract

Aging and age-related neurodegenerative disorders are characterized by the dysfunction or loss of brain nicotinic acetylcholine receptors (nAChRs), and these changes may be related to other senescence markers, such as oxidative stress and DNA repair dysfunction. However, the mechanism of nAChR loss in the aging brain and the modification of this process by drugs (e.g., memantine, Mem) are not yet fully understood. To study whether the differences in nAChR expression in the rat brain occur due to aging or oxidative stress and are modulated by Mem, we analyzed nAChR subunits (at RNA and protein levels) and other biomarkers by real-time quantitative polymerase chain reaction (RQ-PCR) and Western blot validation. Twenty-one female Wistar rats were divided into four groups, depending on age, and the oldest group received injections of Mem or water with the use of intragastric catheters. We studied the cerebral grey matter (CGM), subcortical white matter (SCWM), and cerebellum (Ce). Results showed an age-related decrease of α7 nAChR mRNA level in SCWM. The α7 nAChR mRNA loss was accompanied by reduced expression of 8-oxoguanine DNA glycosylase 1 (OGG1) and an increased tumor necrosis factor alpha (TNFα) level. In the water group, we observed a higher level of α7 nAChR protein in the SCWM and Ce. Biomarker levels changed, but to a different extent depending on the brain area. Importantly, the dysfunction in antioxidative status was stopped and even regressed under Mem treatment. After two weeks of treatment, an increase in TP53 protein level and a decrease in 8-oxo-2′deoxyguanosine (8-oxo-2′dG) level were observed. We conclude that Mem administration may be protective against the senescence process by antioxidative mechanisms.

## 1. Introduction

Aging and age-related neurodegenerative disorders (such as Alzheimer’s and Parkinson’s diseases) are characterized by numerous changes at the molecular level [1,2,3,4,5,6,7,8,9], including the dysfunction or loss of brain nicotinic acetylcholine receptors (nAChRs) [10,11,12,13,14,15]. This decline of nAChR expression could be associated with other senescence markers, such as increased oxidative stress and DNA repair dysfunction [3,5,16,17,18,19]. Some medications, including a drug for Alzheimer’s disease, memantine, can affect nAChRs. However, the mechanism of nAChR loss in the aging brain and the modification of this process by memantine (Mem) are not yet fully understood [8].

Brain-related diseases are characterized by progressive cognitive impairment, severe memory loss, and altered behaviour [15,20,21]. This significantly reduces the quality of life of patients [22,23,24,25]. In the brain of affected individuals, deficits in the cholinergic neurotransmitter system have been reported, with loss of nACh receptors observed in various areas of the brain [26,27]. This is an important change because nAChR receptors appear to be involved in neuroprotection, preventing neuronal death and apoptosis [15,28,29,30]. The main subunits of this receptor (α7, α4, β2) undergo changes during the processes of aging and neurodegeneration [14,29] and are believed to be important in regulating cognitive function [29,31,32]. However, the mechanism of the brain nAChR loss in these neurodegenerative disorders is not fully understood; both increased and decreased nAChR mRNA expression have been reported in several studies [8,33].

Age-related neuronal changes are associated with the formation and accumulation of reactive oxygen species (ROS), which result in oxidative stress that damages all components of the cell (DNA, lipids, and proteins) and could lead to irreversible changes in DNA information and structure [16,18,34,35,36]. DNA oxidative damage relevant to the processes of aging or stress may be estimated by the increased level of the oxidized form of guanine (8-oxo-2′deoxyguanosine) [16,18,34,35,36,37,38]. Importantly, there is a DNA repair system, and the OGG1 and TP53 proteins are part of it [3,16,19,36,37,39,40,41,42,43]. In addition, it is well known that the inflammatory cytokines, e.g., tumor necrosis factor (TNF) superfamily, are linked to oxidative stress [5,44,45,46,47]. It has been shown that the inflammatory response plays a prominent role in pathological brain aging [5,48].

Memantine (Mem) is an uncompetitive antagonist of N-methyl-D-aspartate receptors (NMDARs) (with moderate affinity for the channel) [49,50,51] and has primarily been developed for the treatment of symptoms of Alzheimer’s disease (AD), dementia, and neuropathic pain [52,53,54,55]. Memantine has protective properties in the aging brain and may improve caspase-dependent apoptosis in the aging brain [56,57,58,59,60]. It was demonstrated that Mem blocked α7nAChRs receptors [50,61,62,63].

We hypothesized that the age-related changes in the nAChR expression could be associated with other senescence markers, such as increased oxidative stress and inflammatory process, and could be modified by memantine (Mem) administration. We analyzed RNAs and/or protein expression encoding α7, α4, and β2 nAChR receptors in three brain structures (cerebral grey matter, subcortical white matter, and cerebellum) of young and old rats, as well as in Mem or water groups. In each group, the selected brain areas were also analyzed to determine the markers of oxidative stress (8-oxo-2′dG) and inflammatory (TNF α), as well as two components of the DNA repair system (OGG1 and TP53).

## 2. Results

The study was focused on analyzing comparisons between these groups: (i) old controls (CONTROL II) vs. young controls (CONTROL I), (ii) water group (CONTROL III) vs old controls (CONTROL II), (iii) memantine group vs. water group (CONTROL III). Statistically significant (*p* < 0.05) differences were highlighted. All *p* values were two-tailed and considered statistically significant at a threshold of *p* < 0.05. The trend towards differences was at values of 0.05 < *p* < 0.1.

### 2.1. nAChRs (Nicotinic Acetylcholine Receptors in Brain) Expression Level

#### 2.1.1. α7 Subunit of the nAChR

**Figure 1:**
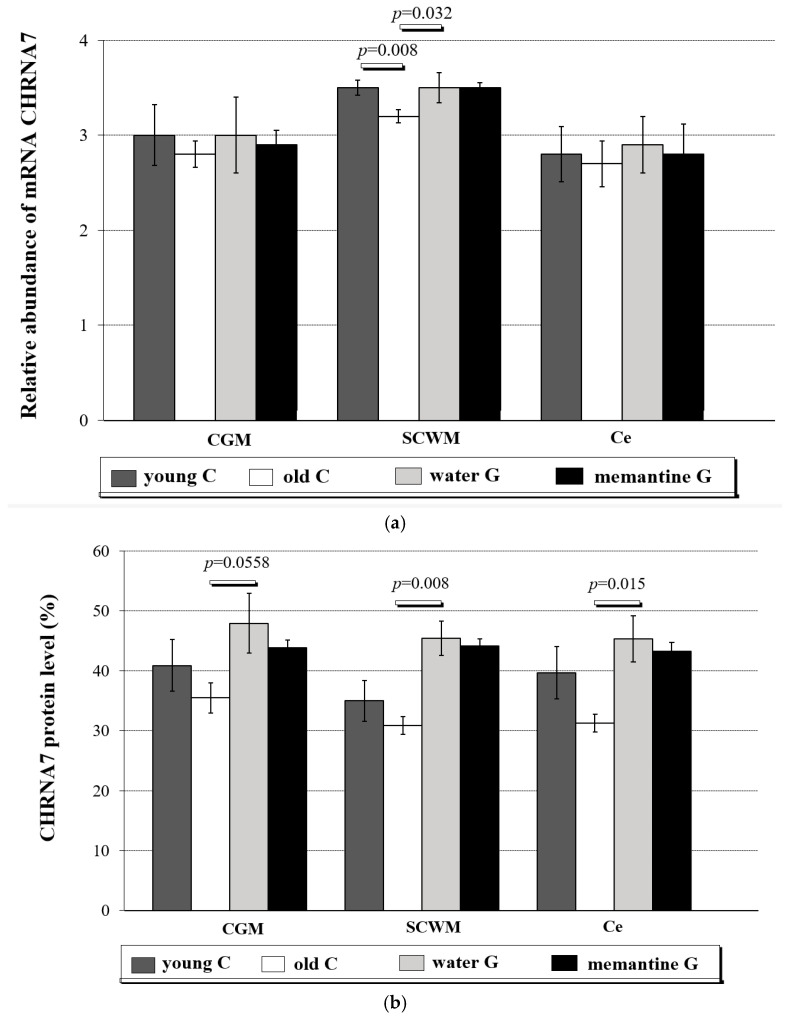

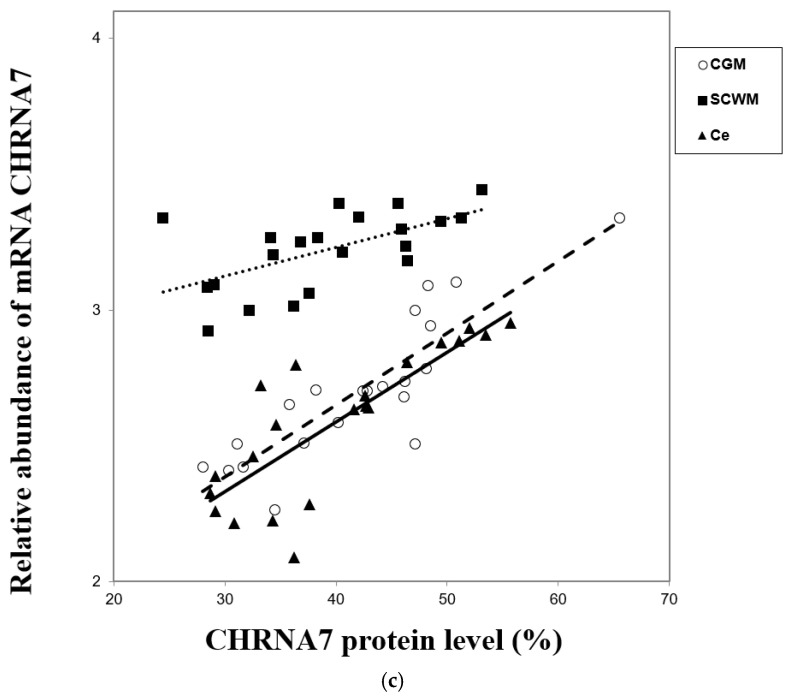
(**a**) Relative abundance of CHRNA7 transcripts in cerebral grey matter (CGM), subcortical white matter (SCWM), and cerebellum (Ce) in four analyzed rat groups (C-controls, G-group)—young C (n = 5); old controls—old C (n = 5); water group—water G (n = 5); memantine group—memantine G (n = 6); all data were expressed as means ± standard deviation (SD); *p*-value for Mann-Whitney test. Explanations: The following three structures were taken from the rats’ brain: cerebral grey matter (CGM) (parietal lobe), subcortical white matter (SCWM) and cerebellum (Ce), and immediately used to isolate RNA, which was reverse-transcribed into cDNA. The relative abundance of mRNA CHRNA7 was evaluated by RQ-PCR analysis. (**b**) CHRNA7 protein level (in % area of immunoreactive bands) in cerebral grey matter (CGM), subcortical white matter (SCWM), and cerebellum (Ce) in four analyzed rat groups: young controls—young C (n = 5); old controls—old C (n = 5); water group—water G (n = 5); memantine group—memantine G (n = 6); all data were expressed as means ± standard deviation (SD); *p*-value for Mann-Whitney test. (**c**) Correlation between relative abundance of CHRNA7 mRNA and CHRNA7 protein levels (in % area of immunoreactive bands) in three analyzed brain regions (cerebral grey matter (CGM), R = 0.868, *p* < 0.0001, subcortical white matter (SCWM), R = 0.597, *p* = 0.0042), and cerebellum (Ce), R = 0.811, *p* < 0.0001); R—coefficient of Pearson or Spearman (for, respectively, parametric or nonparametric data distributions); *p*—level of statistical significance. Explanations: The following three structures were taken from the rats’ brain: cerebral grey matter (CGM) (parietal lobe), subcortical white matter (SCWM), and cerebellum (Ce), and immediately used to isolate RNA, which was reverse-transcribed into cDNA. The relative abundance of mRNA CHRNA7 was evaluated by RQ-PCR analysis.

In the cerebral grey matter (CGM), subcortical white matter (SCWM), and cerebellum (Ce) of young controls, the observed CHRNA7 mRNA levels (mean ± standard deviations) were 3.0 ± 0.32, 3.5 ± 0.08, and 2.8 ± 0.29, respectively (Figure 1a and Supplementary Table S1a).

In SCWM, the mRNA expression level of CHRNA7 was higher in all analyzed groups of animals when compared to Ce and also when compared to CGM in the case of young control, old control, and memantine group (Supplementary Table S1a, in POST HOC test *p* = 0.02 or less).

The expressions of *CHRNA7* in the old control group were CGM 2.8 ± 0.14, SCWM 3.2 ± 0.07, and Ce 2.7 ± 0.24 (Supplementary Table S1a). *CHRNA7* expression was significantly decreased in the SCWM area (Mann-Whitney test, *p* = 0.008) (Figure 1a) in the old control animals in comparison to the young group. In the water group, the level of CHRNA7 mRNA in SCWM was higher than in the old control group (Mann-Whitney test, *p* = 0.032) (Figure 1a and Supplementary Table S1a).

Protein expression levels of CHRNA7 are presented in Figure 1b and Supplementary Table S1b.

The reduction in the level of the α7 nAChR protein was noted in all brain structures of aging rats, and the decreases were 13.2% in the CGM, 11.7% in the SCWM, and 21.2% in the Ce, as compared with young controls (Supplementary Table S1b). In the water group, CHRNA7 protein levels in SCWM and Ce areas were higher as compared to the old control group (Mann-Whitney test, *p* = 0.008 and *p* = 0.015, respectively). A similar trend was observed in the CGM (Mann-Whitney test, *p* = 0.0558) (Figure 1b). We did not observe significant differences in the Mem group in comparison to the water group in all three brain regions, though we noted a trend toward reduced α7 nAChR protein level (*p* = 0.054).

The correlation between CHRNA7 mRNA and α7 nAChR protein expression is presented in Figure 1c and Supplementary Table S1c.

A positive correlation between CHRNA7 mRNA and α7 nAChR protein expression for all analyzed brain regions was observed (CGM R = 0.868, *p* < 0.0001; SCWM R = 0.597, *p* = 0.0042; Ce R = 0.811, *p* < 0.0001) (Figure 1c, details in four analyzed animal groups in Supplementary Table S1c).

#### 2.1.2. α4 and β2 Subunits of the nAChR

**Figure 2:**
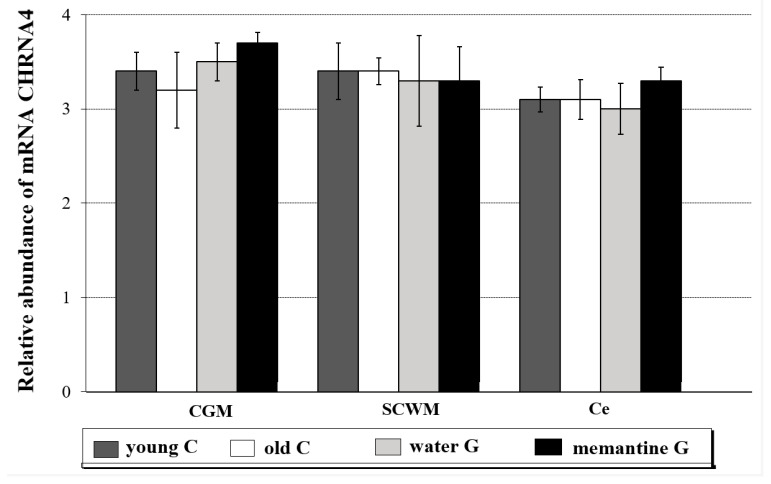
Relative abundance of CHRNA4 transcripts in the cerebral grey matter (CGM), subcortical white matter (SCWM), and cerebellum (Ce) in four analyzed rat groups (C-controls, G-group)—young C (n = 5); old controls—old C (n = 5); water group—water G (n = 5); memantine group—memantine G (n = 6); all data were expressed as means ± standard deviation (SD); *p*-value for Mann-Whitney test. Explanations: The rat brains following three structures were taken: cerebral grey matter (CGM) (parietal lobe), subcortical white matter (SCWM), and cerebellum (Ce), and immediately used to isolate RNA, which was reverse-transcribed into cDNA. The relative abundance of mRNA CHRNA4 was evaluated by RQ-PCR analysis.

The following CHRNA4 mRNA levels were observed in the brain areas analyzed in young animals: CGM 3.4 ± 0.20, SCWM 3.4 ± 0.30, and Ce 3.1 ± 0.14. In the old controls, the water group and the memantine group expressed CHRNA4 similarly (Figure 2 and Supplementary Table S2a).

No significant differences in the expression levels of *CHRNB2* were observed in the different brain regions and tested animal groups (Supplementary Table S2b).

### 2.2. TP53 Protein Level

**Figure 3:**
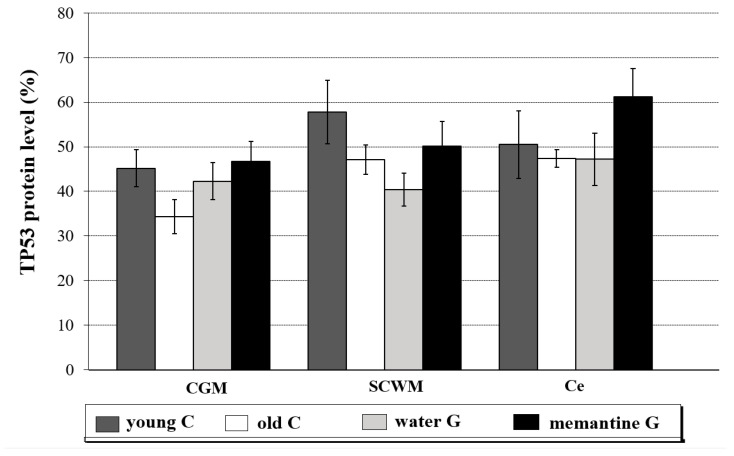
TP53 protein level (in % area of immunoreactive bands) in the cerebral grey matter (CGM), subcortical white matter (SCWM), and cerebellum (Ce) in four analyzed rat groups: young controls—young C (n = 5); old controls—old C (n = 5); water group—water G (n = 5); memantine group—memantine G (n = 6); all data were expressed as means ± standard deviation (SD); *p*-value for Mann-Whitney test.

The results for TP53 protein levels are shown in Figure 3, Supplementary Figure S1 and Supplementary Table S3.

TP53 protein expression in the three analyzed brain structures were similar. The comparison analysis between the young control group and old controls demonstrated no significant differences in TP53 protein levels (Figure 3, Supplementary Table S3). However, in the Mem-treated group, the amounts of TP53 protein were significantly increased in the entire brains of the rats as compared to the water group (Mann-Whitney test; *p* = 0.031, data not presented in figure or table).

### 2.3. OGG1 Expression Level

**Figure 4:**
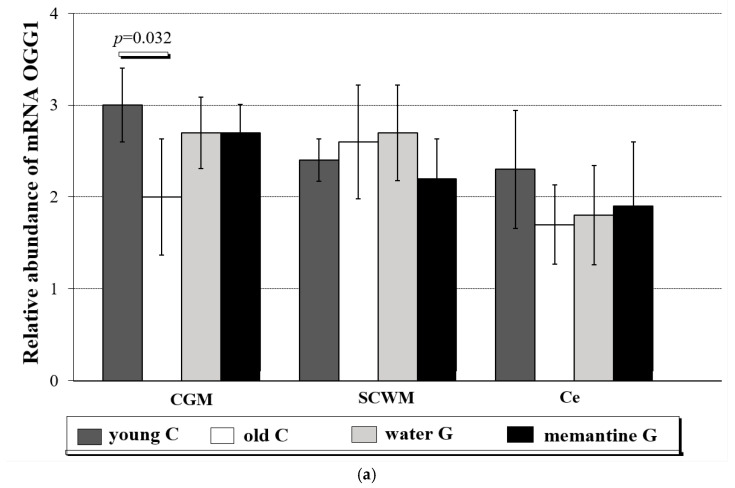

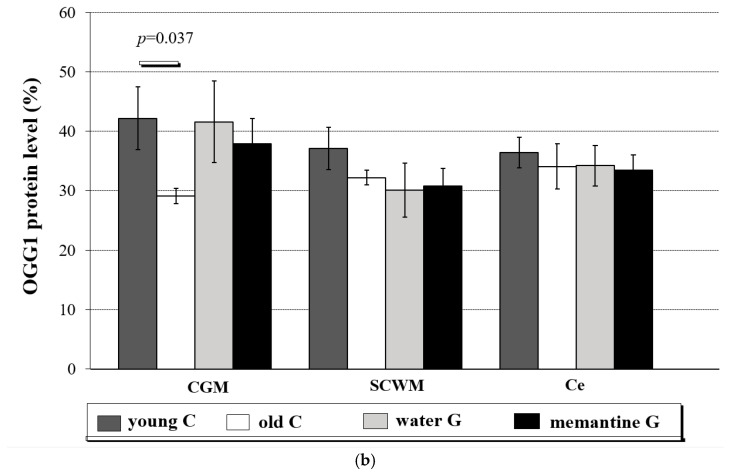
(**a**) Relative abundance of OGG1 transcripts in cerebral grey matter (CGM), subcortical white matter (SCWM) and cerebellum (Ce) in four analyzed rat groups (C-controls, G-group)—young C (n = 5); old controls—old C (n = 5); water group—water G (n = 5); memantine group—memantine G (n = 6); all data were expressed as means ± standard deviation (SD); *p*-value for Mann-Whitney test. Explanations: The rat brains following three structures were taken: cerebral grey matter (CGM) (parietal lobe), subcortical white matter, (SCWM) and cerebellum (Ce), and immediately used to isolate RNA, which was reverse-transcribed into cDNA. The relative abundance of mRNA OGG1 was evaluated by RQ-PCR analysis. (**b**) OGG1 protein level (in % area of immunoreactive bands) in cerebral grey matter (CGM), subcortical white matter (SCWM), and cerebellum (Ce) in four analyzed rat groups: young controls—young C (n = 5); old controls—old C (n = 5); water group—water G (n = 5); memantine group—memantine G (n = 6); all data were expressed as means ± standard deviation (SD); *p*-value for Mann-Whitney test.

The levels of OGG1 mRNA reached 3.0 ± 0.40 in CGM, 2.4 ± 0.23 in SCWM, and 2.3 ± 0.64 in Ce of young controls (Figure 4a and Supplementary Table S4a).

In the old controls, *OGG1* expression was significantly decreased in the CGM (2.0 ± 0.63; Mann-Whitney test, *p* = 0.032) compared to the young controls (Figure 4a). In the entire brains of the aging rats, Western blot analysis revealed a reduction in OGG1 protein levels in old controls compared to young animals (Mann-Whitney test, *p* = 0.014; data not presented in figure or table). This reduction was observed also in the CGM (29.1 ± 1.27; Mann-Whitney test, *p* = 0.037) (Figure 4b). There was no significant influence of water or memantine administration on *OGG1* expression in all tested brain regions (Supplementary Table S4b).

### 2.4. TNFα Protein Level

**Figure 5:**
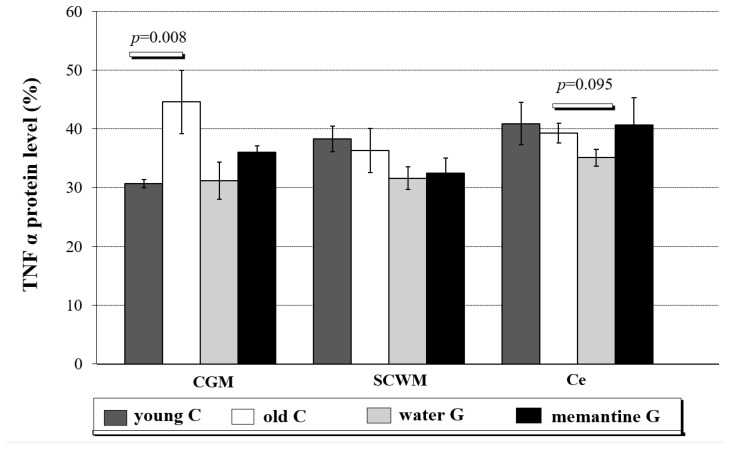
Protein expression levels of TNFα (in % area of immunoreactive bands) in cerebral grey matter (CGM), subcortical white matter (SCWM), and cerebellum (Ce) in four analyzed rat groups: young controls—young C (n = 5); old controls—old C (n = 5); water group—water G (n = 5); memantine group—memantine G (n = 6); all data were expressed as means ± standard deviation (SD); *p*-value for Mann-Whitney test.

The results for TNFα protein levels are shown in Figure 5 and Supplementary Table S5.

TNFα protein levels in the CGM of old controls were higher (44.6 ± 5.37, Mann-Whitney test, *p* = 0.008) than in young controls (CGM 30.7 ± 0.71) (Figure 5 and Supplementary Table S5). TNFα protein level in CGM was also higher than in Ce (POST HOC test, *p* = 0.030) (Supplementary Table S5).

Significant down-regulation in TNFα protein levels was detected in the entire brains of the aging water group when compared to old controls (Mann-Whitney test; *p* = 0.003; data not presented in figure or table). In Ce, a trend to a lower level of TNFα protein levels was observed in the water group as compared with old controls (Mann-Whitney test, *p* = 0.095) (Figure 5 and Supplementary Table S5).

### 2.5. 8-Oxo-2′deoxyguanosine (8-oxo-2′dG) Level

**Figure 6:**
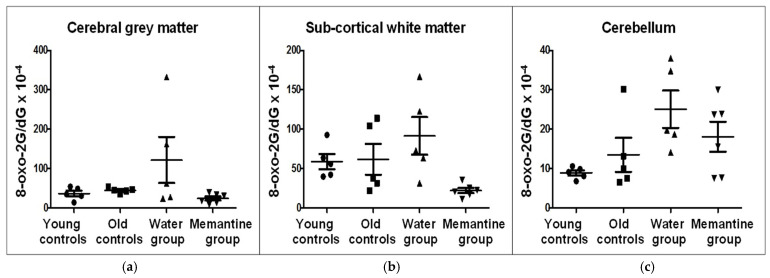
Levels of 8-Oxo-2′dG (8-Oxo-2′deoxyguanosine) in (**a**) cerebral grey matter (CGM), (**b**) subcortical white matter (SCWM), and (**c**) cerebellum (Ce), in four analyzed rat groups: young controls—young C (n = 5); old controls—old C (n = 5); water group—water G (n = 5); memantine group—memantine G (n = 6); The results represent ratios of 8-Oxo2′dG/dG (×10^−4^); dG—deoxyguanosine: *p* = 0.009 for Mann-Whitney test in the case of memantine G vs. water G in SCWM; *p* = 0.056 for Mann-Whitney test in the case of water G vs. old C in Ce.

The analyses for 8-oxo-2′dG levels in the Ce, CGM, and SCWM in four analyzed rat groups are shown in Figure 6 and Supplementary Table S6.

It was found that the levels of 8-oxo-2′dG in the Ce were lower (8.9 ± 1.50) than in the CGM (36.8 ± 15.86) and SCWM (58.7 ± 21.33) of young controls (POST HOC test *p* = 0.03 and *p* = 0.0007, respectively). In old controls, the levels of 8-oxo-2′dG were also lower in the Ce than in the SCWM (13.5 ± 9.62 in the Ce, 44.7 ± 6.89 in the CGM, and 61.8 ± 43.65 in the SCWM, POST HOC test *p* = 0.030) (Figure 6 and Supplementary Table S6).

A trend to an increase in 8-oxo-2′dG levels was detected in the Ce of water-treated animals, compared to old controls (Mann-Whitney test; *p* = 0.056). Mem administration significantly reduced the levels of 8-oxo-2′dG in the entire brain of the aging rats when compared to water-treated animals (Mann-Whitney test; *p* = 0.002, data not presented in figure or table), mostly in the SCWM (Mann-Whitney test; *p* = 0.009) (Figure 6 and Supplementary Table S6).

### 2.6. Correlation Between TP53 and TNFα Protein Levels

TP53 was found to positively correlate with TNFα in the SCWM of young controls (R = 0.980, *p* = 0.003, Supplementary Table S7, Supplementary Figure S2). A positive correlation trend was also observed in the Ce of young controls (R = 0.811, *p* = 0.096). A similar, positive correlation trend between TP53 and TNFα proteins in the SCWM was also noted for the water group (R = 0.875, *p* = 0.052, Supplementary Table S7).

### 2.7. Correlation Between OGG1 and TP53 Protein Levels

A negative correlation between the OGG1 and TP53 protein levels in the Ce of water group animals was observed (R = −0.914, *p* = 0.030, Supplementary Figure S3, Supplementary Table S8). However, a positive correlation trend was observed in the SCWM in old controls (R = 0.854, *p* = 0.065) (Supplementary Table S8).

### 2.8. Correlation Between OGG1 Protein and 8-Oxo-2′dG Levels

The level of 8-oxo-2′dG in the Ce of old controls showed a trend of positive correlation with the OGG1 protein level (R = 0.844, *p* = 0.072, Supplementary Table S9). No relationship was observed in the other studied brain areas and groups of animals.

### 2.9. Correlation Between TNFα Protein, 8-Oxo-2′dG Levels, and OGG1 mRNA

It was found that the TNFα protein level positively correlated with the level of 8-oxo-2′dG (R = 0.788, *p* = 0.017, Supplementary Figure S4a) in the Ce of old controls. Treatment of old rats with Mem resulted in a negative correlation between these two proinflammatory markers in the SCWM (R = −0.891, *p* = 0.017, Supplementary Table S10, Supplementary Figure S4b).

Moreover, there was a positive correlation between TNFα protein and the OGG1 mRNA in the CGM in the young controls (R = 0.900, *p* = 0.037, Supplementary Figure S5a), with a negative correlation in old controls (R = −0.900, *p* = 0.037, Supplementary Table S11, Supplementary Figure S5b).

### 2.10. Correlation Between α7 nAChR and TP53 Proteins Levels

In the CGM of young controls, there was a positive correlation between protein levels of TP53 and α7 nAChR (R = 0.917, *p* = 0.028, Supplementary Table S12, Supplementary Figure S6). There was no correlation between these parameters in other analyzed animal groups and brain areas.

### 2.11. Correlation Between α7 nAChR and OGG1 Transcript Levels

In young controls, mRNA level of α7 nAChR in the SCWM negatively correlated with the levels of OGG1 mRNA (R = −0.897, *p* = 0.039, Supplementary Figure S7a). α7 nAChR mRNA also negatively correlated with levels of OGG1 mRNA in the SCWM of the old controls (R = −0.949, *p* = 0.014, Supplementary Figure S7b). Moreover, in the water group, a positive correlation was observed between the α7 nAChR mRNA and the OGG1 mRNA levels in the CGM (R = 0.885, *p* = 0.046) (Supplementary Table S13, Supplementary Figure S7c).

### 2.12. Correlation Between α4 and β2 nAChRs, and OGG1 Transcript Levels

α4 nAChR transcript level and OGG1 mRNA level was positively correlated (R = 0.961, *p* = 0.0009, Supplementary Figure S8a) in the Ce of young controls. Similarly, a positive correlation was observed between α4 nAChR mRNA level and OGG1 mRNA level in the CGM of old controls (R = 0.884, *p* = 0.046, Supplementary Table S14, Supplementary Figure S8b). Additionally, it was found that α4 nAChR transcript levels negatively correlated with β2 nAChR transcript levels in the Ce of young animals (R = −0.883, *p* = 0.047, data not presented in figure or table). A negative correlation was observed also between β2 nAChR mRNA and OGG1 mRNA levels in Ce of young controls (R = −0.900, *p* = 0.037) (Supplementary Table S15, Supplementary Figure S9).

### 2.13. Correlation Between α7, α4, and β2 nAChRs Transcript and 8-Oxo-2′dG Levels

In young controls, the protein level of α7 nAChR in the SCWM negatively correlated with the level of 8-oxo-2′dG (R = −0.887, *p* = 0.045, Supplementary Table S16, Supplementary Figure S10). There was also a negative correlation between the 8-oxo-2′dG level and α4 nAChR transcript levels in the SCWM of Mem-treated animals (R = −0.829, *p* = 0.042) (Supplementary Table S17, Supplementary Figure S11).

The levels of 8-oxo2′dG and β2 nAChR mRNA were negatively correlated (R = −0.946, *p* = 0.015, Supplementary Figure S12) in the CGM of the water group. However, after two weeks of Mem administration, we observed a trend for positive correlation in the level of β2 nAChR transcript with 8-oxo-2′dG (R = 0.784, *p* = 0.065) in the CGM (Supplementary Table S18).

## 3. Discussion

Aging (and age-related neurodegenerative diseases) is characterized by dysfunction or loss of neuronal nicotinic acetylcholine receptors (nAChR) in various areas of the brain [6,10,11,12,13,14,15,26,27]. These nACh receptors play a key role in modulating central nervous system (CNS) function and in the development of various brain pathologies [28,64]. However, the mechanism of nAChR loss in the brain in these neurodegenerative disorders is not yet fully understood.

In the present experimental study, we evaluated the associations between oxidative stress in aging rat brains, dysfunction of oxidative DNA damage repair, cholinergic receptor expression, and the role of Mem in reducing the effects. We found that brain processes associated with aging were associated with a decrease in the expression of nAChR receptor α7 (at the RNA level but not protein), without statistical changes in the case of mRNA nAChR receptors α4 and β2. We found increased expression of markers of oxidative stress (8-oxo-2′dG) and inflammation (TNFα), as well as changes in the expression of two markers of the DNA repair system (OGG1 and TP53). These molecular markers of aging can be modified after the administration of memantine.

We studied the cerebral grey matter (CGM), subcortical white matter (SCWM), and cerebellum (Ce). The white matter is essential for transmitting information in the neural network between different brain regions. Alterations at the level of the white matter lead to disrupted connections in subcortical-cortical areas, which are responsible for cognitive and executive functions, information processing, and memory. The white matter is also involved in cognitive impairment, as are the grey matter and cerebellum [65,66].

Aging is a natural process of progressive decline in organismal function, and the brain is the most sensitive and vulnerable to this process. The senescence of the human brain is accompanied by moderate dendritic, synaptic, and axonal degeneration with nearly no cell loss [67,68,69,70]. Moderate degenerative changes have been observed in the cholinergic neurons of the basal forebrain complex, which have been associated with progressive age-related memory deficits. This cholinergic hypofunction has also been observed in neurodegenerative disorders like AD and is associated with dysfunction or loss of nAChRs, which modulate major cognitive CNS functions, including learning and memory formation. As a result of such changes, one can observe an alteration in the expression of cellular signal transduction genes [12,13,71,72].

### 3.1. nAChR Expression (α7, α4, and β2 Subunits), Senescence, and the Role of Memantine

We analyzed the expression of *CHRNA7*, *CHRNA4*, and *CHRNB2* in young and aged rats. A comparison between these two groups revealed that the expression of *CHRNA7* was lower in the SCWM in old animals compared to young animals. Interestingly, the SCWM demonstrated the highest level of *CHRNA7* expression compared to the other analyzed brain structures. Our data confirmed previously published results showing that normal aging is characterized by a decline in *CHRNA7* expression in humans and mice [73]. The main (and important for regulating cognitive function) subtypes of nAChR modulated by aging and neurodegeneration are α7, α4, and β2 subunits [14,29,31,32]. Loss of nACh receptors has been observed in various brain areas [6,26,27]. The results of our analysis of the expression of the α4 subunit were similar in different brain areas (CGM, SCWM, and Ce) both in young and old female animals. The results of our study are in agreement with previously published data by Charpantier et al. [74]. They demonstrated no change in α4 mRNA expression in eight analyzed brain structures in aging male rats [74]. Another study showed the down-regulation of α4 mRNA expression in the frontal cortex of aged humans and unchanged α4 mRNA levels in the remaining brain areas [75,76]. Results about the expression of CHRNA4, reported by Ferrari et al. [76], detected an age-related decrease in α4 subunit expression in older rat brains. The α4 mRNA expression level showed a decrease of 20–30% at 29 months of age and, in some areas, reached 50% at 32 months. It is possible that our old animals (18–24 months) were too young to observe statistically significant changes in the α4 mRNA expression level. As reported in our work, the results for β2 mRNA expression levels were similar to those obtained for the α4 nAChR subunit, probably because the co-assembly of α4 with β2 nAChR subunits results in the formation of functional receptors.

The results of our study have also revealed that the injection of water can lead to a change in nAChR expression in old rats. Interestingly, the protein level of α7 nAChR was up-regulated in SCWM and Ce after water injection compared to aged controls. We did not observe significant differences in the Mem group in comparison to the water group in all three brain regions, though we noted a trend to reduced α7 nAChR protein level (*p* = 0.054). These results are partly similar to the data obtained by other groups, e.g., Marvanová et al. [77], who used cDNA microarray analysis to identify 28 genes regulated by Mem in the adult rat brains and did not report altered expression of nAChRs. The study of Motawaj et al. also demonstrated no changes in the mRNA expression of CHRNA7 after Mem treatment [78]. The observed trend to a decrease in α7 nAChR protein levels in our study may be explained by different times of Mem administration in analyses. Marvanová et al. analyzed genes regulated by Mem after a single dose of Mem [77], whereas in the study of Motawaj et al. the expression of α7 nAChR was studied two days after a 5-day memantine (Mem) treatment [78]. In our study, however, Mem was administered over a greater period of time (two weeks) and in a 2-fold higher dose to detect its potential effect on nAChRs expression.

Interestingly, in the literature, a close correlation has been found between high levels of lipid peroxidation and a decrease in the number of nicotinic receptors in AD brains [79,80]. Significant increases in the total number of astrocytes and in the number of astrocytes expressing the α7 nAChR subunit, along with significant decreases in the levels of α7 and α4 nAChR subunits on neurons, were observed in the hippocampus and temporal cortex of both APPswe (Swedish amyloid precursor protein) and sporadic AD brains [79]. Furthermore, the number of α7 nAChR binding sites in the temporal cortex of the APPswe brain was significantly lower than in the younger control group, reflecting a decreased neuronal level of α7 nAChR. These findings suggest different regulatory mechanisms and roles of the α7 nAChR in astrocytes and neurons. In another study by Yu et al., an increase in the expression level of α7 nAChR in astrocytes was positively correlated with the extent of neuropathological alterations, especially the number of neuritic plaques, in the AD brain [81]. The elevated expression of α7 nAChR in astrocytes might participate in the A-beta cascade and the formation of neuritic plaques, potentially playing an important role in the pathogenesis of AD. Since astrocytes can release glutamate in response to specific stimulation [82], at least in vitro, which may induce a release of homocysteic acid (HCA) [83] that forms homocysteine (Hcy), the latter being an excitotoxicity factor and endogenous agonist for the brain N-methyl-D-aspartate receptors (NMDARs, which play a key role in brain function, development, and health) [84,85]. Because of this, Hcy is considered to be a risk factor for both vascular and neurodegenerative diseases, such as AD [86,87,88,89,90]. Literature reports, as well as our previous investigations [37], indicate that the development of AD has been accompanied by increased 8-oxo-2′dG and Hcy levels. Interestingly, as we reported in this study, the anti-oxidative influence of Mem, being the most spectacular in the SCWM, might be because of its Hcy-antagonistic properties for NMDA receptors.

### 3.2. Oxidative DNA Damage (8-Oxo-2′dG Marker), DNA Repair System (OGG1 and TP53), and Memantine

One of the hallmarks of DNA modification in cells undergoing oxidative stress is 8-oxo-2′dG, considered to be a marker of DNA damage in aging processes as well as in degenerative disorders [16,18,34,36,37,38]. We previously confirmed the presence of oxidative damage to DNA in experimental animals [37], and similarly to the results obtained in that report, our present investigations demonstrate that SCWM is the most sensitive to oxidative DNA injury, independent of the animal’s age. SCWM of the CNS, which consists of myelinated axons and glial cells, with astrocytes being the most abundant type of the latter, is more vulnerable to oxidative injury. It is possible that the augmented sensitivity of the white matter to reactive oxygen species (ROS) reflects its biochemical composition, which consist of mainly lipids, which are particularly sensitive to ROS action, and the aldehydes formed due to lipid peroxidation may induce DNA injury. Several reports have demonstrated the presence of oxidative stress markers in age-related diseases like dementia or AD [16,18,34,35,36,91,92]. In the previous study, we also suggested that oxidative DNA damage increases only during the early stages of AD and then decreases with the progression of the disease due to the activation of a compensatory system [35]. Studies of human brains indicate that the level of 8-oxo-2′dG is higher in mitochondrial than in nuclear DNA in aging older individuals [4,93]. Guan et al. have suggested that oxidative stress is an early event in AD and is likely to play a more active role in the pathogenesis than previously suggested [80]. Our earlier reports have confirmed this hypothesis [35,36].

Reports from the literature have demonstrated that the levels of damaged DNA is associated with aging [94,95] and were doubled in two-year-old rats as compared to young animals [96]; however, Fraga et al. did not detect an increase in DNA damage with increased age in the entire brains of old rats [97]. In that study, it is possible that oxidative DNA injury increased in different stages of life, mainly in stress situations, and may involve only one specific brain structure. Our previous laboratory experiments on the animal model [98] have demonstrated that the levels of 8-oxo-2′dG were increased in the whole brains of old rats, but they were significantly higher only in the Ce as compared to young controls. In the present study, Ce demonstrated the lowest level of 8-oxo-2′dG compared to the CGM in young control and old control groups. It is probable that the level of oxidative damage to DNA also reflects the affected CNS region. It may be that Purkinje cells, the largest neurons of the CNS present at the margins of the granular zone of the cerebellar cortex, exhibit particular sensitivity to oxidative stress (hypoxia and ischemia) [99,100,101]. However, the protective mechanisms, whereby the oxidative injury to DNA in the Ce of aged rats in normal and stress conditions is relatively low, are also responsible for the minimal effect of oxidative stress on the Ce. The relatively low level of 8-oxo-2′dG detected in the Ce (compared especially to SCWM) in young and old controls in our study may be linked to a specific form of the organism’s protection against oxidative injury of this brain structure. Mem administration significantly reduced the levels of 8-oxo-2′dG in the entire brain of the aging rats when compared to the water group (*p* = 0.002). Antioxidant properties of Mem derivatives have been recently studied, and those studies have revealed a significant neuroprotective activity of Mem against oxidative stress in vitro [52,102,103,104,105].

The level of oxidative DNA injury in the course of aging may also reflect the efficiency of specific enzymes of the DNA repair system [3,16,19,36,37,39,40,41,42,43]. The decreased activity of the enzyme OGG1 was documented both in human natural aging and in precocious aging in mice [35,36,39,95]. It is believed that along with the increase in oxidatively altered bases in DNA, the weakening of the OGG1 glycosylase enzymatic repair system of oxidized guanine occurs both in physiological aging processes and in AD [106,107]. However, as demonstrated in our previous report [36], in particular, when looking at the stage of AD during which the disease progressed from mild to moderate dementia, there was an increase in the OGG1 protein level along with the increase of oxidative DNA damage. In the present study on an animal model, the old controls demonstrated a reduction in *OGG1* expression in CGM compared to young individuals.

The role of TP53 in a DNA repair system has also been observed [36,41,42,43,108]. The ability of TP53 to enhance the activity of OGG1 for the removal of 8-oxo-2′dG was demonstrated in vitro and in vivo [109,110]. Our present investigations showed a positive correlation trend between the OGG1 and TP53 protein levels in the SCWM in the old control group, but a negative correlation between these protein levels was observed in the Ce of the water group. Moreover, in the Mem-treated group, the amounts of TP53 protein were significantly increased in the entire brains of the rats compared to the water group (*p* = 0.031). Interestingly, the results of Zhang et al. and of Ioudina and Uemura also showed that the TP53 protein level increased in cultured human cells, as well as in rat neurons and astrocytes in the stress culture condition, such as culture in the presence of amyloid beta-peptide [111,112].

There have been reports indicating that the TP53 protein participates in neuronal apoptosis in the brain of AD patients, and an increased expression of the *TP53* gene has been observed in the brain of AD patients as well as in the peripheral blood lymphocytes (PBLs) of patients with AD [34,35,36]. A negative relationship between the TP53 and OGG1 protein levels in the Ce of the water group supports our previous findings about an inverse correlation between these two DNA repair proteins in the PBLs of AD patients [36]. We have previously suggested that an increased level of the TP53 protein in patients between the ages of 62 and 83 years and without dementia features is involved in the repair of oxidative DNA damage rather than in neuronal degeneration [36]. In this report [36], we also demonstrated that after the 60th year of life, the TP53 protein levels remained higher in each stage of AD development, with a tendency to decrease as dementia features progressed. According to reports in the literature and our earlier investigations, the highest levels of 8-oxo-2′dG and TP53 occur in the early stages of AD and then tend to decrease during the neurodegenerative process of developing dementia, probably due to oxidative modifications leading to changes in the activity of the TP53 protein [34,113].

### 3.3. Biomarker Correlations, Aging, and Brain Areas, and the Effects of Memantine (Mem)

It has been shown that TNFα may induce both pro-apoptotic and anti-apoptotic responses in neuronal cells. In vitro studies have shown that TNFα, as a proinflammatory factor, promotes cell death by apoptosis in the neuronal cell line [114,115,116]. However, there are also studies indicating that TNFα action is transduced through binding to TNF receptor I and can promote both cell death and the survival of cells [117,118,119]. It has been shown that the inflammatory response plays a prominent and early role in pathological brain aging, and a significant increase of proinflammatory cytokines (IL-1, IL-6, TNFα, and IL-8) and their receptors have been reported [120]. Published genome-wide association studies (GWAS) have identified genes that are involved in inflammation and associated with an increased risk of developing AD [121,122,123,124]. In addition, it has been demonstrated that TNFα plays an important role in the development of AD neuro-inflammation, leading to increased oxidative stress and impairment of cognitive function [125,126,127,128]. Our earlier report also demonstrated that TNFα protein levels were significantly decreased in humans over 60 years old as compared to younger control individuals, and that the decreased TNFα level was linked to a reduced OGG1 level [36]. It is possible that the reduced level of OGG1 is important for the immune response in the aging process and may also be associated with a decreased risk of diseases of old age. Studies by Mabley et al. [129] and Touati et al. [130], carried out on infected OGG1−/− deficit mice, demonstrated that inactivation of the OGG1 enzyme increased the protective effect from damages and reduced the inflammatory response due to a marked decrease in the production of proinflammatory cytokines such as TNFα [129,130,131]. In the present study, significant down-regulation in TNFα protein levels was detected in the entire brains of the water group when compared to old controls (*p* = 0.003). At the same time, we showed that exposing the ageing animals to the stress of an intragastric catheter administration of water did not mobilize their body to increase TNFα protein levels, which could subsequently lead to activation of the apoptosis pathway. TNFα protein levels in the CGM of old controls were higher (*p* = 0.008) than in young controls. In the Ce, a trend to lower levels of TNFα protein in the water group as compared with old controls was also observed (*p* = 0.095). Moreover, decreasing TNFα levels were found to correlate with decreasing TP53 levels in the SCWM of water-treated animals. We also demonstrated that high levels of TNFα protein correlated with high OGG1 mRNA levels in the CGM of young animals and old controls.

The results of our study suggest that nAChRs could play a protective role in oxidative DNA damage in the brain. This would be in accordance with the proper role of nAChRs in forming the cholinergic neurotransmitter system, deficits of which are often observed in individuals affected by age-related brain diseases with impaired learning and memory functions. Thus, in young controls, a negative correlation between mRNA of *CHRNA7* and OGG1 mRNA, as well as a negative correlation between CHRNA7 protein and 8-oxo-2′dG levels, was observed in the SCWM. Additionally, in this group of animals, a positive correlation was observed between CHRNA4 mRNA and OGG1 mRNA in the Ce. In the SCWM of aged controls, we found a positive correlation between the CHRNA4 and OGG1 mRNA levels. In the present study, we also observed a positive correlation between levels of the CHRNA7 and TP53 proteins in the CGM of young controls. These results have confirmed our previous animal studies [98], which indicated that the expression of the *TP53* gene in different brain regions increased with the age of experimental rats.

Moreover, we found that the amounts of TP53 were significantly increased in the entire brains of the Mem-treated rats compared to water-treated animals. These data suggest that Mem could be implicated in antioxidative mechanisms either directly or indirectly by increasing the level of TP53 protein. The observed correlations between nAChRs and the DNA repair system support our previous report about the putative relationship between the *CHRNA4* genetic polymorphisms and 8-oxo-2′dG level, as well as the TP53 protein levels, in the PBLs of AD patients [34]. However, the relationship between other nAChRs, the levels of 8-oxo-2′dG, and the TP53 protein had not been reported before.

Because of elevated α7 subunits of the nAChR brain expression after water injections, we were able to demonstrate that a protective role of the cholinergic compensatory system in the aging brain exists and is efficient mainly at the early stages of oxidative DNA damage. However, it may be insufficient in organisms undergoing prolonged exposure to stressful environmental conditions.

Despite many advantages, our study has some limitations related to the analysis of only female animals. Hence, our observations are only specific to female rats and require confirmation in a male animal population. In addition, more extended experiments than 14 days may be connected with more changes in the subunits of nAChRs brain expression. Longer analyses and studies involving a larger group of rats (due to the relatively large scatter in study results) would be advisable in the future. It is noteworthy that the results obtained from in vivo animal model studies are more helpful for assessing pathomorphological and pathophysiological changes in people than in vitro animal model studies. However, as with any study conducted on animals (rats), it is not recommended to transfer them to clinical outcomes directly.

## 4. Materials and Methods

### 4.1. Reagents

Memantine (Ebixa^®^) was obtained from Lundbeck SAS (Paris, France).

### 4.2. Animals

The animal experimental protocols were approved by the local Ethics Review Committee and carried out in accordance with the guidelines for the care and use of laboratory animals (Ethics Committee of Poznan University of Life Sciences, protocol no. 74/2007, 21 December 2007). All rats were born and bred in our own colony and housed under the same conditions. The animals were fed a standard laboratory chow and were allowed free access to tap water.

Twenty-one adult female Wistar strain rats were divided into four experimental groups: (1) young controls—young C; (2) old controls—old C; (3) water group—water G; (4) memantine group—memantine G. (1) The first group consisted of five 3.0–3.5 month-old females weighing approximately 250 g at the time of brain structure isolation, which was assigned as a young control group (young controls—young C, n = 5, CONTROL I). The remaining sixteen females aged 18–24 months, weighing 300–400 g, were divided into the three following groups: (2) an aged control group of 5 rats (old controls—old C, n = 5, CONTROL II); (3) a group of 5 rats with administered water by intragastric catheter in a volume of 0.5 mL/kg body weight once per day, for two weeks (water group—water G, n = 5, CONTROL III); (4) memantine-treated group of 6 rats (memantine group—memantine G, n = 6). This last group received memantine (Mem) by intragastric catheter at a dose of 20 mg/kg body weight (dissolved in water) once per day for two weeks (in a volume of 0.5 mL/kg body weight).

The animals were kept in a constant laboratory environment at 21 °C throughout the study. After light anesthesia with 5% halothane (Narcotan, Zentiva, Prague, Czech Republic) by inhalation in a glass chamber (no other pharmacological agents were administered), rats were sacrificed by decapitation 24 h after the last dose of memantine or water. In all animal groups after decapitation, heads were immersed in liquid nitrogen and stored at −80 °C (24 h) for further analysis. Before the next step, the frozen heads were transferred to a freezer with a temperature of −20 °C (24 h) and then into the fridge (4 °C). Afterwards, the rat brains were cooled (stored in dry ice), the following three structures were taken: cerebral grey matter (CGM), subcortical white matter (SCWM), and cerebellum (Ce), using a surgical scalpel and manual methods.

### 4.3. Tissue Preparation for Western Blot

For Western blot analysis, the brain tissues were rinsed with Radio Immunoprecipitation Assay (RIPA) buffer (50 mM Tris-HCl, pH 7.2, 150 mM NaCl, 1% IGEPAL CA-630, 0.05% SDS, and 1% sodium deoxycholate) supplemented with protease inhibitor cocktail for mammalian cell extracts (Sigma-Aldrich, St. Louis, MO, USA). The tissue was then manually homogenized in a mixture of RIPA and protease inhibitor cocktail (16:1) in the presence of phenylmethylsulfoxyl fluoride (PMSF) (Sigma-Aldrich, St. Louis, MO, USA).

The homogenate rat brain structures were centrifuged at 12,000× *g* for 10 min at 4 °C. The total protein content in the obtained supernatant was established, according to Lowry et al. [132].

### 4.4. Western Blot

Protein samples were subjected to electrophoresis in a polyacrylamide gel. The α7 (CHRNA7), OGG1, and TNFα proteins were analyzed in 12% polyacrylamide gel and the TP53 protein in 7.5% polyacrylamide gel (Supplementary Figure S1). Equivalent amounts of proteins were loaded into the wells (30 µg/lane). The gel-separated proteins were electrotransferred to a nitrocellulose filter in a semidry Western blot analysis apparatus (Massy Cedex, Apelex, France). For protein detection, the following primary antibodies (Abs) (all from Santa Cruz Biotechnology, Dallas, TX, USA) were used: anti-CHRNA7 goat polyclonal Ab (sc-1447, C-20), anti-OGG1/2 goat polyclonal Ab (G-20, IgG), anti-TNFα goat polyclonal Ab (N-19, IgG), and anti-TP53 mouse monoclonal Ab (IgG-2a). All Abs were diluted at 1:500. Then, individual sheets of nitrocellulose filter were incubated with the secondary Ab (all from Santa Cruz Biotechnology, Dallas, TX, USA). The following secondary Abs, all diluted at 1:2000, were used: for CHRNA7 protein, donkey anti-goat horseradish peroxidase (HRP)-conjugated Ab, for OGG1 and TNFα proteins, mouse anti-goat IgG-HRP Ab, and for TP53, protein goat anti-mouse IgG-HRP Ab. To stain immune-reactive bands, peroxidase BMB was added (BM blue POD substrate precipitation, Roche Diagnostics GmbH, Mannheim, Germany). The surface area of the immune-reactive bands was registered using a densitometer (GS-710; Bio-Rad, Hercules, CA, USA) in the Quantity One System, as previously described in detail by Dorszewska et al. [98,133].

All animal samples from one group were loaded on the same gel in duplicate with a pooled sample of all groups as a reference, allowing for comparison between groups. All samples were standardized to the pooled reference sample and normalized to glyceraldehyde-3-phosphate dehydrogenase (*GAPDH*).

### 4.5. Real-Time Quantitative Polymerase Chain Reaction (RQ-PCR)

Three structures of rat brains (CGM, SCWM, and Ce) were immediately used to isolate RNA, which was reverse-transcribed into cDNA. The relative abundance of CHRNA7, CHRNA4, CHRNB2, OGG1 mRNAs was evaluated by real-time quantitative polymerase chain reaction (RQ-PCR) analysis. The total polyA+ mRNA from each brain structure was isolated according to the method of Chomczynski and Sacchi [134]. Total RNA was extracted from the samples with TRI Reagent (Sigma, St Louis, MO, USA) and RNeasy MinElute cleanup Kit (Qiagen, Hilden, Germany). The RNA samples were treated with DNase I and quantified. The amount of total mRNA was determined from optical density at 260 nm, and the RNA purity was estimated using the 260/280 nm absorption ratio (higher than 1.8) (NanoDrop spectrophotometer, Thermo Fisher Scientific, Madison, WI, USA). The RNA integrity and quality were evaluated using a Bioanalyzer 2100 (Agilent Technologies, Inc., Santa Clara, CA, USA). The resulting RNA integrity numbers (RINs) were between 8.5 and 10, with an average of 9.2 (Agilent Technologies, Inc., Santa Clara, CA, USA). RNA in each sample was diluted to a concentration of 100 ng/µL with an OD260/280 ratio of 1.8/2.0.

Reverse transcription (RT) into cDNA was performed in the presence of an RNase inhibitor using the First Strand cDNA Synthesis kit (Roche Diagnostics GmbH, Mannheim, Germany). The RT-PCR reactions were carried out for 1 μg of brain-derived total RNA in a total volume of 20 μL. For quantification of the obtained cDNA, RQ-PCR was performed in a LightCycler real-time PCR detection system (Roche Diagnostics GmbH, Mannheim, Germany) with software version 4.05. To ensure accuracy and reliability in the quantification process, the expression of the housekeeping GAPDH gene was used as an internal control, serving as a reference gene for normalization. The complete gene sequences to design primers for the amplification of the CHRNA4, CHRNA7, CHRNB2, OGG1, and GAPDH genes were downloaded from the Rat Genome Database (RGD) (http://rgd.mcw.edu) [135].

The primers used in RQ-PCR were designed with the Primer 3 software (Whitehead Institute for Biomedical Research, Cambridge, MA, USA). The primers were designed to span two adjoining introns to eliminate possible contamination of DNA amplification interfering with mRNA quantification. The relative expression levels of *CHRNA4*, *CHRNA7*, *CHRNB2*, and *OGG1* were analyzed using the following pairs of primers: *CHRNA4-F* tgacacgggcagtagaagg and *CHRNA4-R* ccagaaggcagacaatgatg, *CHRNA7-F* ctgaagtttgggtcctggtc and *CHRNA7-R* atctgggtatggctctttgc, *CHRNB2-F* tgcgaagtgaggatgatgac and *CHRNB2-R* acggtcccaaagacacagac, *OGG1-F* ggacctggttttagcttct and *OGG1-R* tagagctgatcctcggtctga. The primers were purchased from the Laboratory of DNA Sequencing and Oligonucleotide Synthesis (Institute of Biochemistry and Biophysics, Polish Academy of Sciences, Warsaw, Poland).

With the above-mentioned primers the SYBR^®^ Green I was used, as a detection dye system. For amplification, 2 μL of template cDNA solution (standard or control) was added to 18 μL of the LightCycler FastStart DNA Master SYBR Green I mix (Roche Diagnostics GmbH, Mannheim, Germany) with previously determined optimal MgCl_2_ concentration (3.5 µM for one reaction), in the presence of primers at a final concentration of 0.3 μmol/L. The real-time PCR program included a 10-min denaturation step to activate the Taq DNA Polymerase, followed by a three-step amplification program: denaturation, annealing, and extension. The specificity of the reaction products was checked by the determination of melting points (0.1 °C/s transition rate).

The results of mRNA expression encoding genes were quantified in relation to the expression of the housekeeping GAPDH gene, which was used as an internal control. GAPDH mRNA serves as a reference gene for normalization and is used frequently in differential gene expression analysis by RT-qPCR [136]. This method provides a more precise measure of gene expression differences and allows for the comparison of gene expression levels in different samples under various experimental conditions. This strategy also effectively reduces the risk of false-positive amplification signals, which could interfere with measuring specific mRNA levels.

### 4.6. Determination of 8-Oxo-2′dG

#### 4.6.1. Isolation of DNA

DNA was isolated from three previously partitioned brain structures (cerebral grey matter, subcortical white matter, and cerebellum) by incubation (24 h, 37 °C) in a lysis buffer containing 10 mM Tris-HCl pH 8.2, 400 mM NaCl, and 2 mM Na_2_EDTA in the presence of sodium dodecyl sulfate (SDS, 10%) and solution of proteinase K (Sigma, USA) (0.1% proteinase K, 10% SDS, 0.1 M Na_2_EDTA) in 100:7:17 proportion [137]. The next step was adding concentrated NaCl solution (6 M) in 1:2 proportion. The cell lysate was centrifuged, and the DNA, present in the upper layer, was precipitated by pouring cold 98% ethanol slowly down the wall of the tube (in proportion 1:2). The last reagent was used after cooling for 30 min at −20 °C in the freezer. The isolated DNA was dissolved in a solution of 0.01 M NaOAc pH 4.5. DNA purity was tested on a 1.0% agarose gel with ethidium bromide. Electrophoretic separation of the DNA matrix was carried out on a 1% agarose gel electrophoresis kit (Kucharczyk Electrophoretic Techniques Ltd., Warsaw, Poland) containing Tris-borate EDTA (TBE) buffer (0.045 M Tris-HCl with boric acid and 0.01 M Na2EDTA pH 8.0). 

#### 4.6.2. Quantification of 8-Oxo-2′dG

In order to determine the 8-oxo-2′dG level, 0.5 U DNA was hydrolyzed to nucleosides by 5 µm P1 nuclease (Sigma-Aldrich, St. Louis, MO, USA) for 2 h at 37 °C. The solution was supplemented with 100 mM Tris-HCl (pH 7.5), to adjust the pH of the solution to 7.5. Subsequently, the DNA was subjected to decomposition using alkaline phosphatase (1 U/µL, Roche Diagnostics GmbH, Mannheim, Germany) for 1 h at 37 °C. The obtained nucleotide mixture was analyzed by HPLC/UV system (P580A, Gynkotek, Gemering, Germany) coupled to an electrochemical detector (CoulArray 5600, ESA, Chelmsford, MA, USA). Nucleosides were separated in Termo Hypersil BDS C18 (250 × 4.6 × 5 µm) column (Dreieich, Germany). The data were collected and processed using Chromeleon software version 4.32 (Gynkotek, Gemering, Germany). The results were presented as a ratio of oxidized nucleosides in the form of 8-oxo-2′dG to unmodified dG (deoxyguanosine).

### 4.7. Statistical Analysis

All data were expressed as means ± standard deviation (SD). When two groups of animals were compared (e.g., water group vs memantine group), we used a test for two independent groups (i.e., the Mann-Whitney test). In the cases comparing more than two groups (such as comparing three brain regions within one animal group), an ANOVA test and the corresponding POST HOC when comparing more than two groups (such as comparing three brain regions within one animal group). POST HOC Tukey test was used for parametric and POST HOC Dunn test—for nonparametric data distributions. Normal data distribution was proved using the Shapiro-Wilk test. Using the Levene test, we checked whether several groups had the same variance in the population.

The correlation between the two obtained results for each individual brain structure was assessed by using the Pearson correlation or the Spearman rank correlation test (for, respectively, parametric or nonparametric data distributions). They were employed to evaluate the positive or negative correlation between two continuous variables in a given group. A positive correlation was represented by the correlation coefficient R value between 0.00 and 1.00 (where as one variable decreased, the other variable decreased; and vice versa), while R = 0.00 indicated no correlation, and R having a value between -1.00 and 0.00 (a contrary relationship between two variables) indicated a negative (also known as inverse) correlation (where as one variable increased, the other variable decreased).

The programs GraphPad Prism 6 (InStat, San Diego, CA, USA) and Statistica 10.0 for Windows (StatSoft, Tulsa, OK, USA) were used to perform statistical analysis on the results. All *p* values were two-tailed and considered statistically significant at a threshold of *p* < 0.05. The trend towards differences was at values of 0.05 < *p* < 0.1 (but this was not statistically significant).

## 5. Conclusions

In this study, we demonstrated an age-related decrease of α7 nAChR expression and the role of oxidative stress in the rat brain. Additionally, we were able to demonstrate that a protective role of the cholinergic compensatory system in the aging brain exists and is efficient mainly at the early stages of oxidative DNA damage. In our study, we report that the brain processes related to aging, such as an increased expression of oxidative and inflammatory biomarkers, could be modified after memantine administration. These data suggest that Mem slows down or protects against the inevitable senescence process by antioxidative mechanisms, but the observed changes have a different extent depending on the area of the brain. Whether these protective effects of Mem are directly connected with NMDA receptors could be evaluated in future studies.

## Data Availability

All relevant data are presented in the manuscript and Supplementary Materials.

## Supplementary Materials

The following supporting information can be downloaded at: https://www.mdpi.com/article/10.3390/ijms26041634/s1.
