# Supplementary material for: Expression of Neuronal Nicotinic Acetylcholine Receptor and Early Oxidative DNA Damage in Aging Rat Brain—The Effects of Memantine
Source: Int J Mol Sci. 2025 Feb 14;26(4):1634. doi: 10.3390/ijms26041634 (PMC11855568; doi:10.3390/ijms26041634)
Supplement: Supplementary file 1 [file ijms-26-01634-s001.zip › Supplementary Figures S1-S12.pdf]

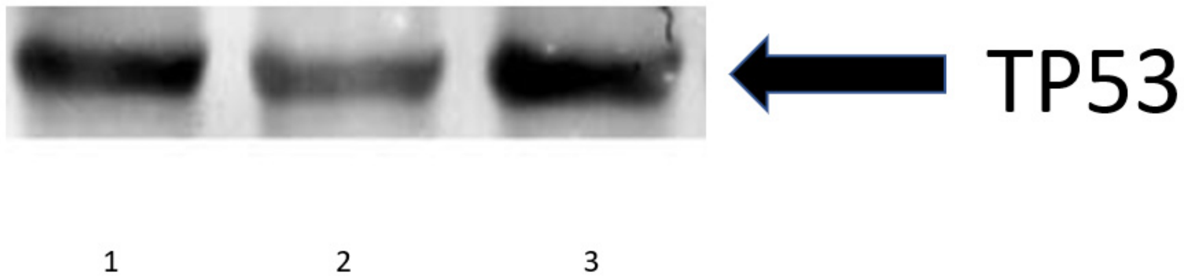

Figure S1.
Western blot analysis of TP53 protein level.
1-3 in 24-month-old rat (1—cerebral grey matter (CGM), 2—subcortical white matter (SCWM), 3—cerebellum (Ce)).

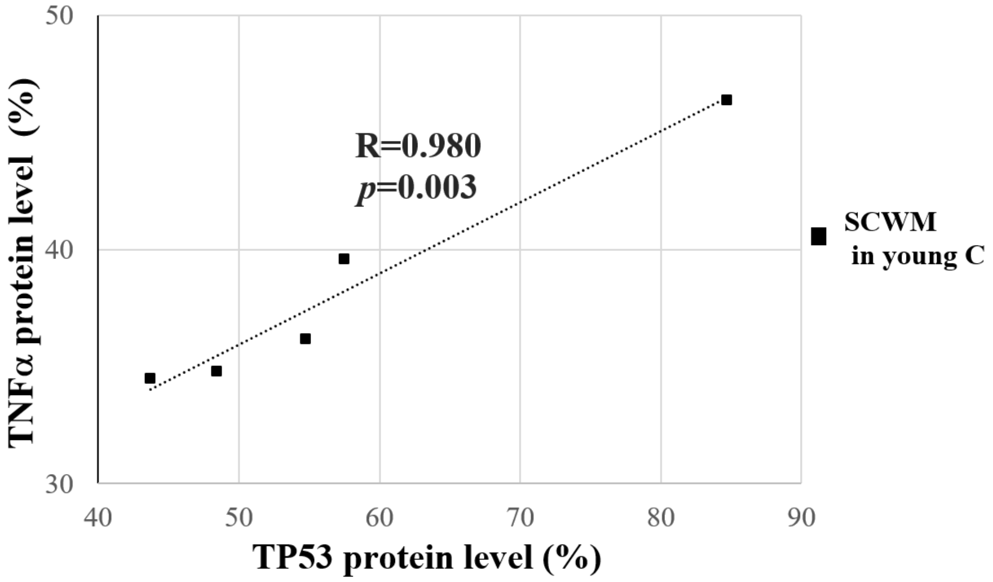

Figure S2.
Correlation between TP53 protein and TNFα protein levels (in % area of immunoreactive bands) in subcortical white matter (SCWM) of young control (young C)
R—coefficient of Pearson or Spearman (for, respectively, parametric or nonparametric data distributions), *p*—level of statistical significance

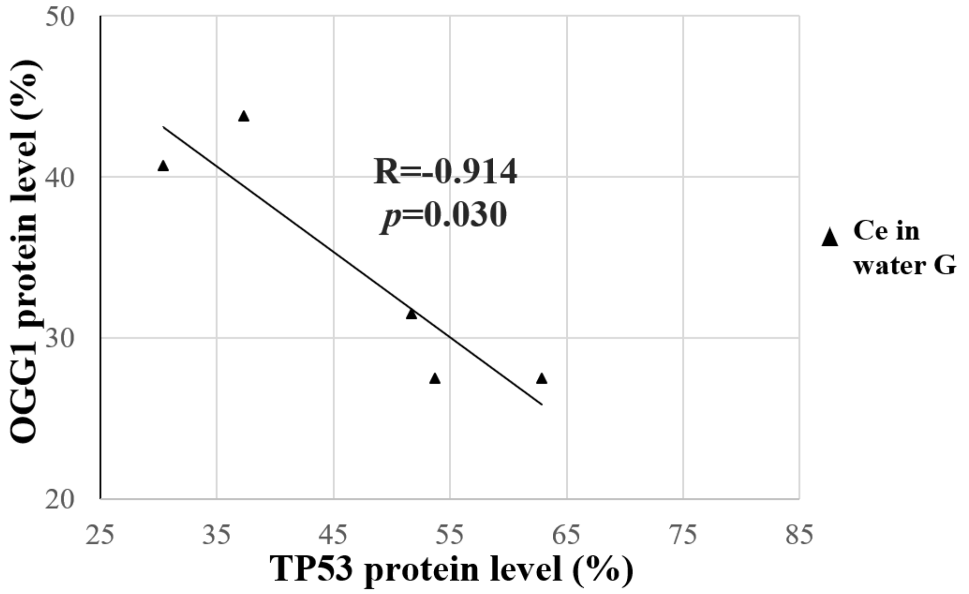

Figure S3.
Correlation between TP53 protein and OGG1 (8-oxoguanine DNA glycosylase 1) protein levels (in % area of immunoreactive bands) in cerebellum (Ce) of water group (water G)
R—coefficient of Pearson or Spearman (for, respectively, parametric or nonparametric data distributions), *p*—level of statistical significance

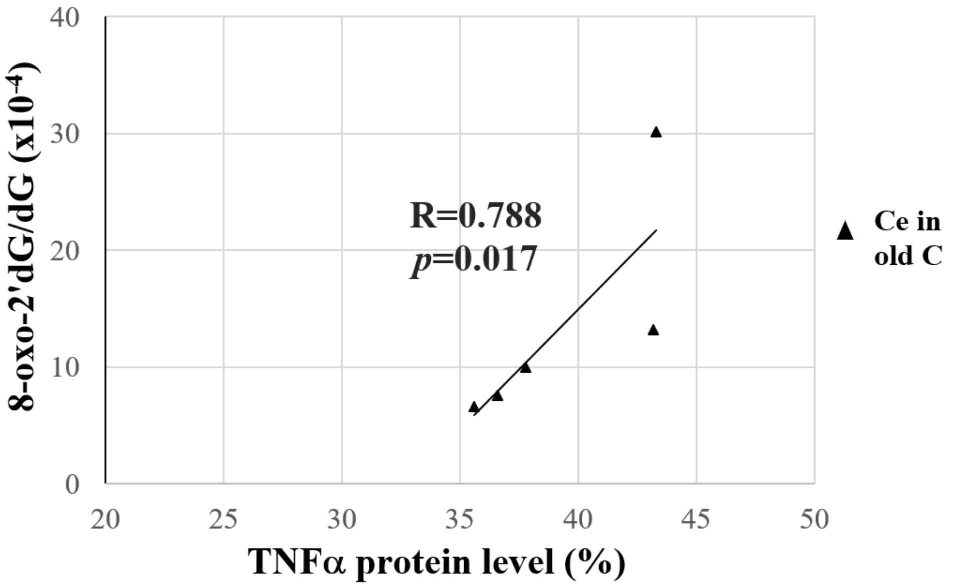

Figure S4a.
Correlation between TNFα protein (in % area of immunoreactive bands) and 8-oxo-2′dG (8-oxo-2′deoxyguanosine) levels in cerebellum (Ce) of old control (old C)
R—coefficient of Pearson or Spearman (for, respectively, parametric or nonparametric data distributions), *p*—level of statistical significance, dG—deoxyguanosine

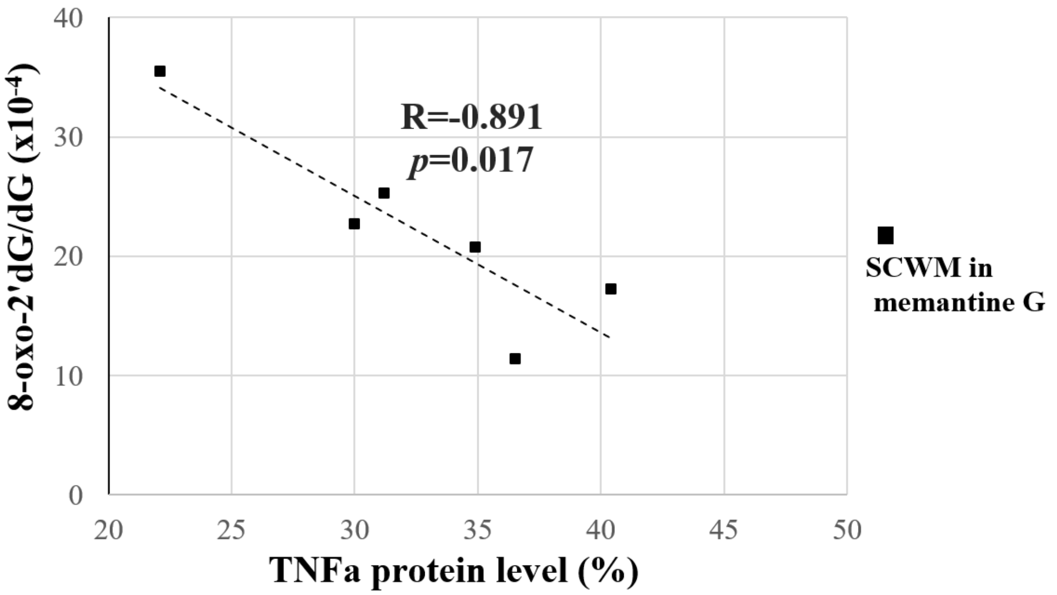

Figure S4b.
Correlation between TNFα protein (in % area of immunoreactive bands) and 8-oxo-2’dG (8-oxo-2’deoxyguanosine) levels in subcortical white matter (SCWM) of memantine group (memantine G)
R—coefficient of Pearson or Spearman (for, respectively, parametric or nonparametric data distributions), p—level of statistical significance, dG - deoxyguanosine

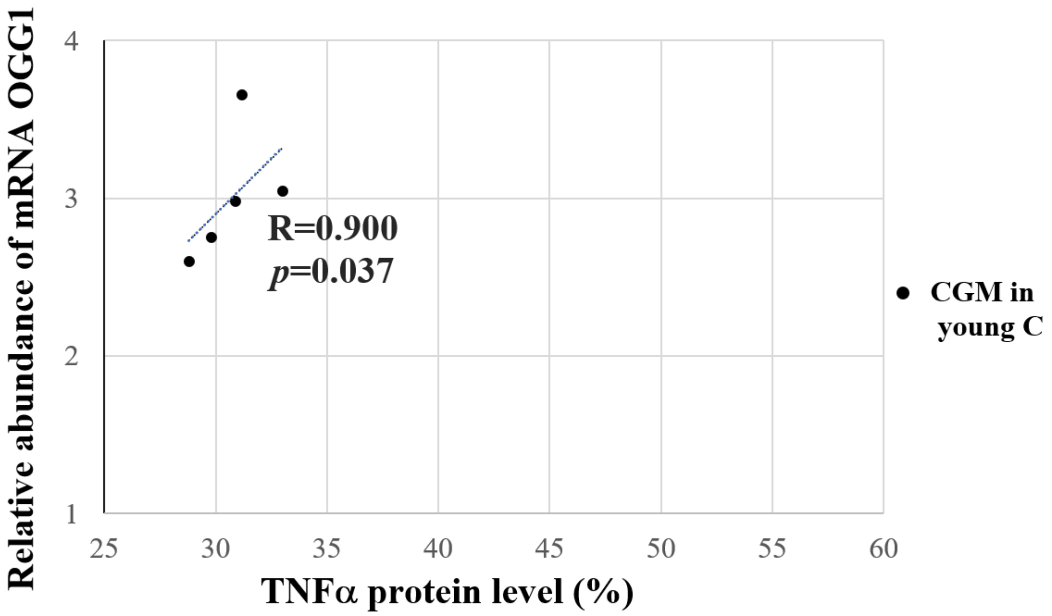

Figure S5a.
Correlation between TNFα protein (in % area of immunoreactive bands) and relative abundance of mRNA OGG1 (8-oxoguanine DNA glycosylase 1) levels in cerebral grey matter (CGM) of young control (young C)
R—coefficient of Pearson or Spearman (for, respectively, parametric or nonparametric data distributions), *p*—level of statistical significance

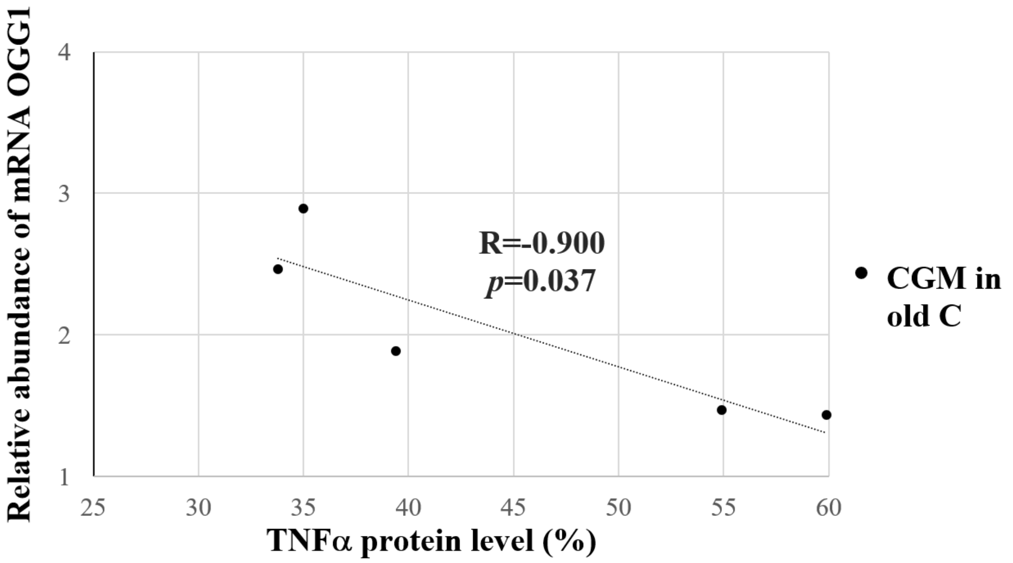

Figure S5b.

Correlation between TNFα protein (in % area of immunoreactive bands) and relative abundance of mRNA OGG1 (8-oxoguanine DNA glycosylase 1) levels in cerebral grey matter (CGM) of old control (old C)

R—coefficient of Pearson or Spearman (for, respectively, parametric or nonparametric data distributions), *p*—level of statistical significance

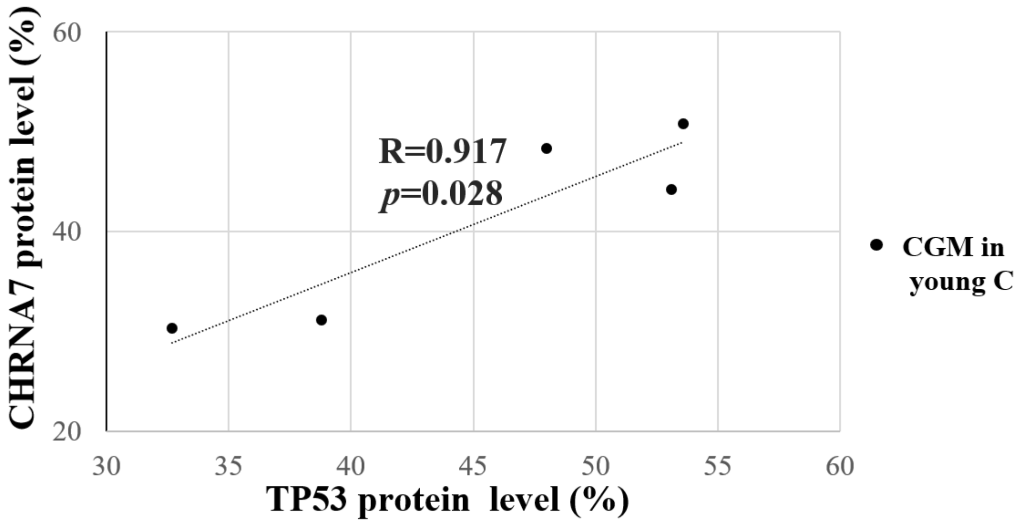

Figure S6.

Correlation between TP53 protein and CHRNA7 protein levels (in % area of immunoreactive bands) in cerebral grey matter (CGM) of young control (young C)

R—coefficient of Pearson or Spearman (for, respectively, parametric or nonparametric data distributions), *p*—level of statistical significance

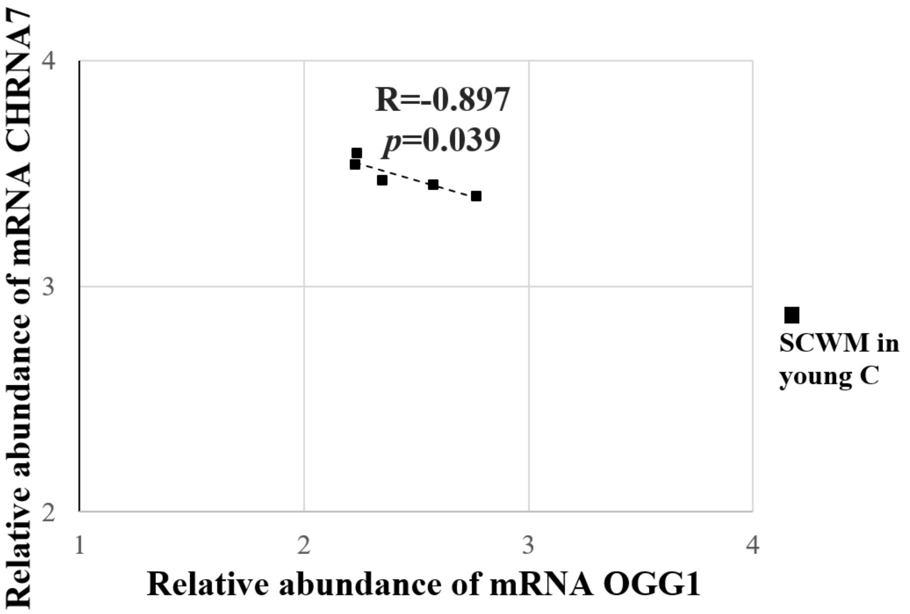

Figure S7a.
Correlation between relative abundance of mRNA OGG1 (8-oxoguanine DNA glycosylase 1) and relative abundance of mRNA CHRNA7 levels in subcortical white matter (SCWM) of young control (young C)
R—coefficient of Pearson or Spearman (for, respectively, parametric or nonparametric data distributions), *p*—level of statistical significance

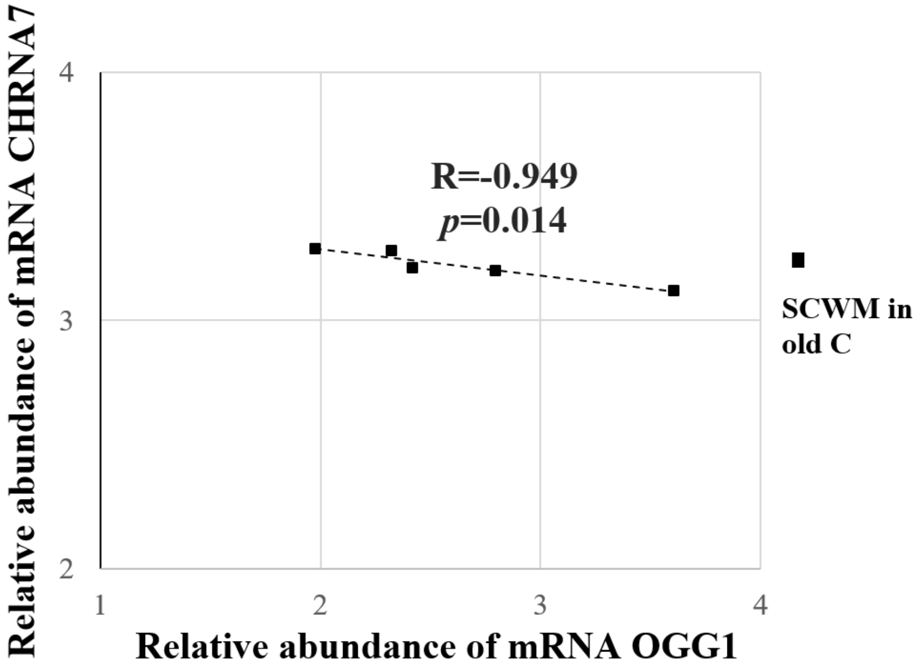

Figure S7b.
Correlation between relative abundance of mRNA OGG1 (8-oxoguanine DNA glycosylase 1) and relative abundance of mRNA CHRNA7 levels in subcortical white matter (SCWM) of old control group (old C)
R—coefficient of Pearson or Spearman (for, respectively, parametric or nonparametric data distributions), *p*—level of statistical significance

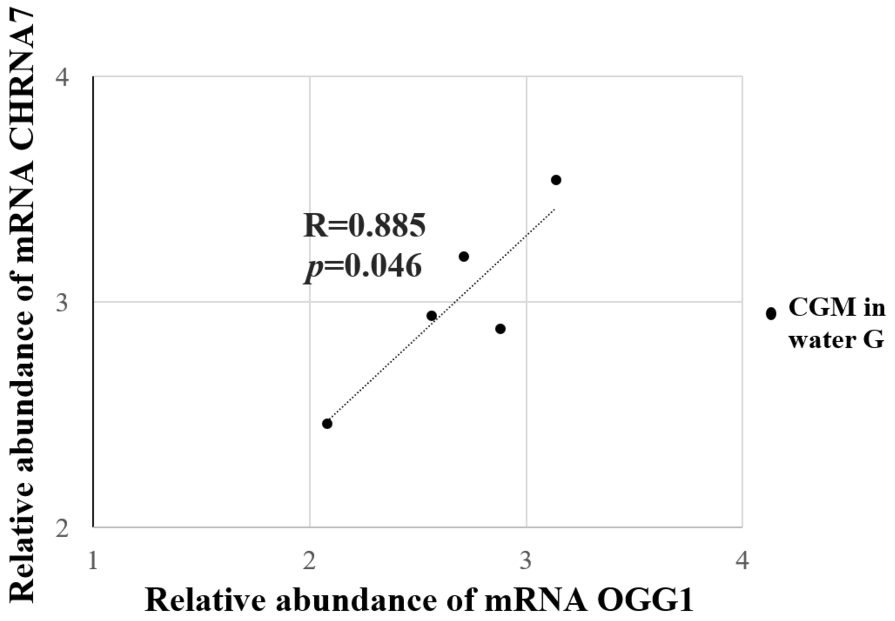

Figure S7c.

Correlation between relative abundance of mRNA OGG1 (8-oxoguanine DNA glycosylase 1) and relative abundance of mRNA CHRNA7 levels in cerebral grey matter (CGM) of water group (water G)

R—coefficient of Pearson or Spearman (for, respectively, parametric or nonparametric data distributions), p—level of statistical significance

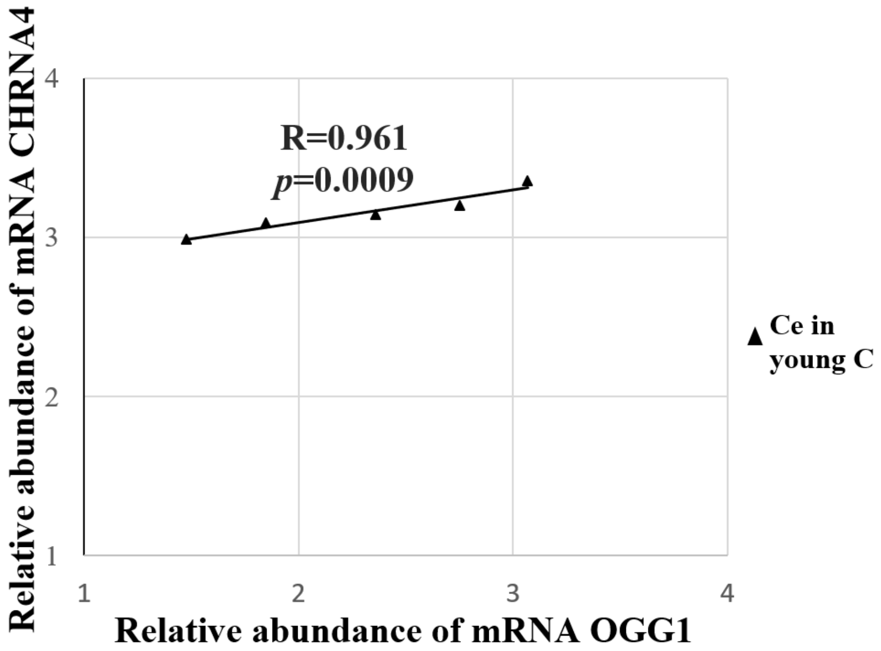

Figure S8a.

Correlation between relative abundance of mRNA OGG1 (8-oxoguanine DNA glycosylase 1) and relative abundance of mRNA CHRNA4 levels in cerebellum (Ce) of young control (young C)

R—coefficient of Pearson or Spearman (for, respectively, parametric or nonparametric data distributions), p—level of statistical significance

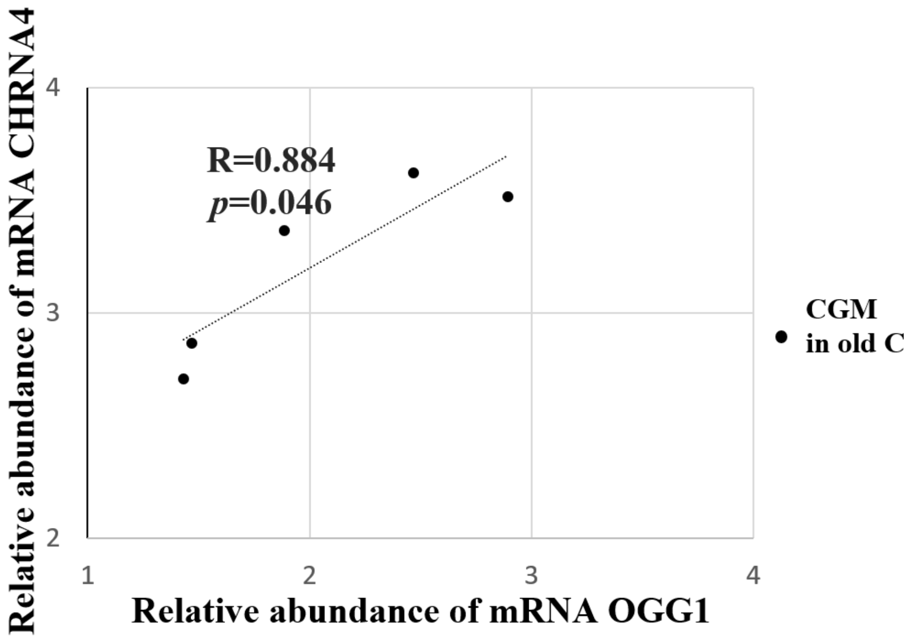

Figure S8b.
Correlation between relative abundance of mRNA OGG1 (8-oxoguanine DNA glycosylase 1) and relative abundance of mRNA CHRNA4 levels in cerebral grey matter (CGM) of old control (old C)
R—coefficient of Pearson or Spearman (for, respectively, parametric or nonparametric data distributions), p—level of statistical significance

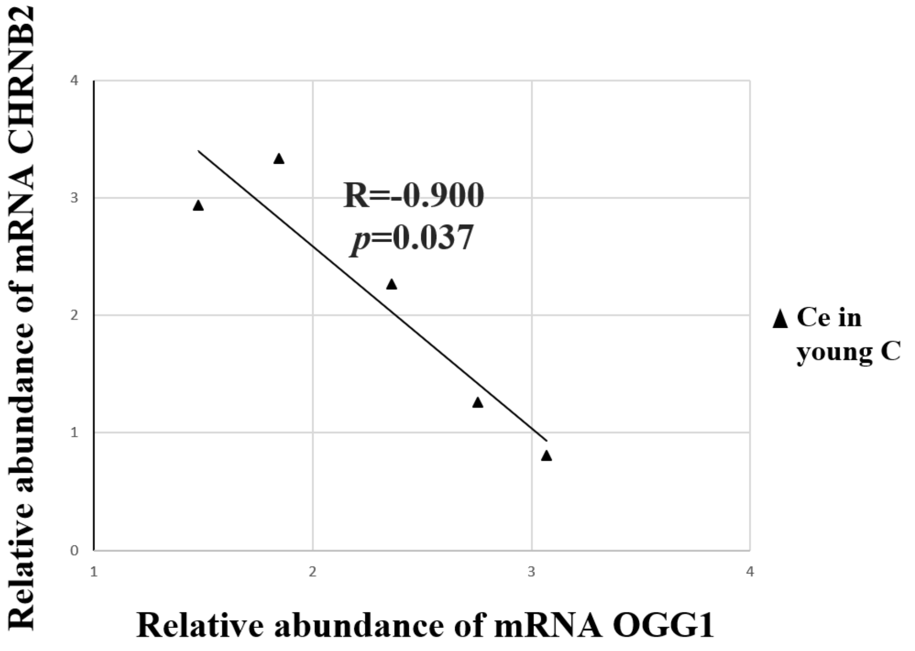

Figure S9.
Correlation between relative abundance of mRNA OGG1 (8-oxoguanine DNA glycosylase 1) and relative abundance of mRNA CHRNB2 levels in cerebellum (Ce) of young control (young C)
R—coefficient of Pearson or Spearman (for, respectively, parametric or nonparametric data distributions), *p*—level of statistical significance

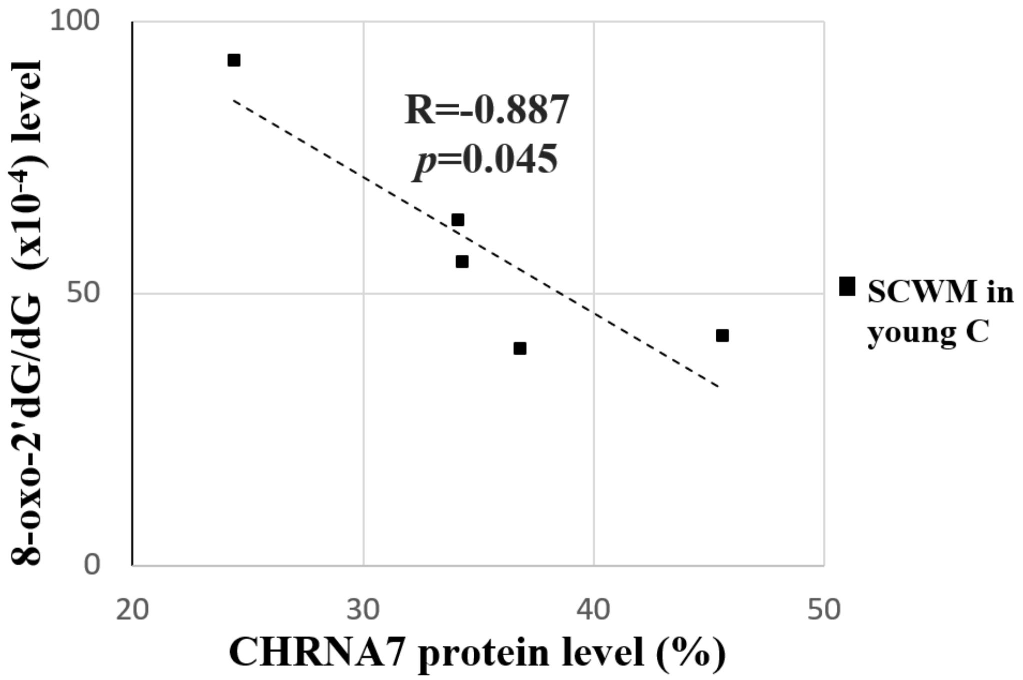

Figure S10.
Correlation between CHRNA7 protein (in % area of immunoreactive bands) and 8-oxo-2′dG (8-oxo-2′deoxyguanosine) levels in subcortical white matter (SCWM) of young control (young C)
R—coefficient of Pearson or Spearman (for, respectively, parametric or nonparametric data distributions), *p*—level of statistical significance, dG – deoxyguanosine

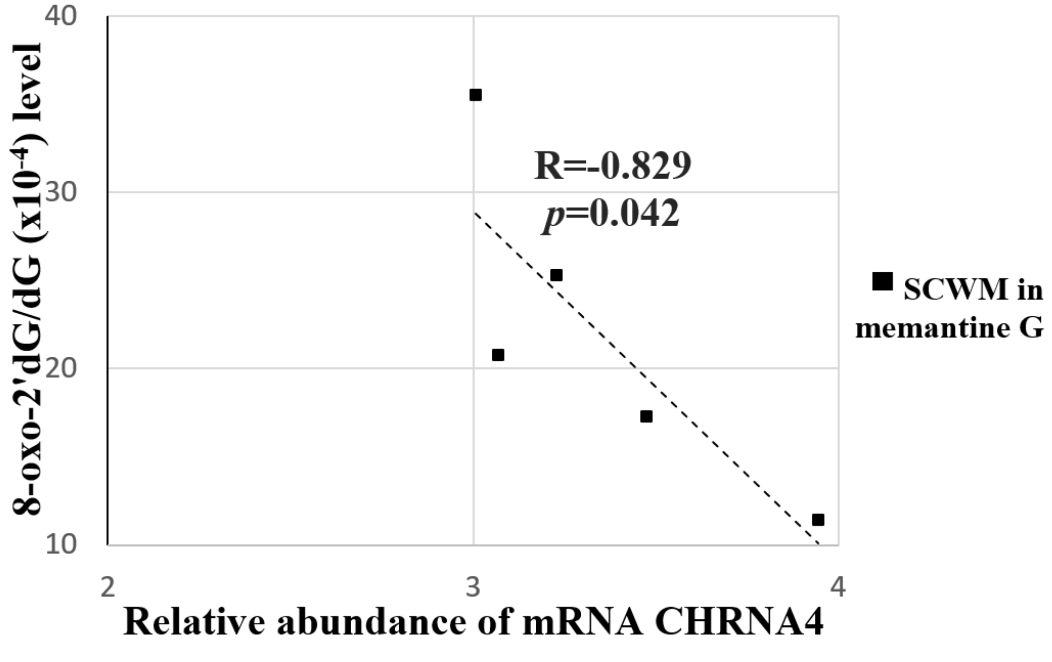

Figure S11.
Correlation between relative abundance of mRNA CHRNA4 and 8-oxo-2′dG (8-oxo-2′deoxyguanosine) levels in subcortical white matter (SCWM) of memantine group (memantine G)
R—coefficient of Pearson or Spearman (for, respectively, parametric or nonparametric data distributions), *p*—level of statistical significance, dG – deoxyguanosine

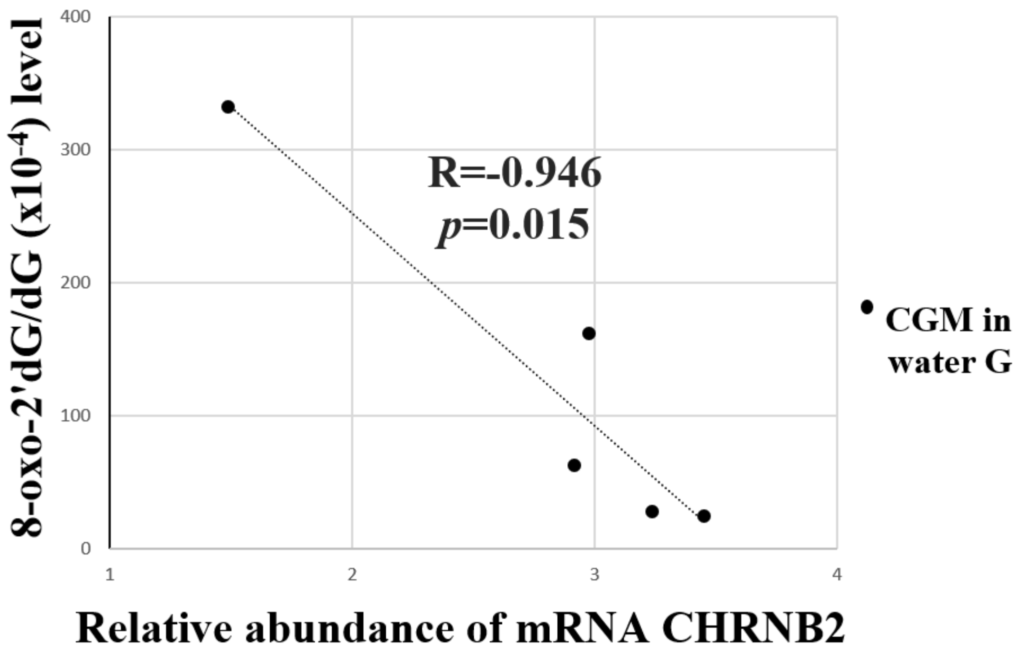

Figure S12.

Correlation between relative abundance of mRNA CHRNB2 and 8-oxo-2′dG (8-oxo-2′deoxyguanosine) levels in cerebral grey matter (CGM) of water group (water G)
R—coefficient of Pearson or Spearman (for, respectively, parametric or nonparametric data distributions), *p*—level of statistical significance, dG – deoxyguanosine
