# Supplementary material for: Expression of Neuronal Nicotinic Acetylcholine Receptor and Early Oxidative DNA Damage in Aging Rat Brain—The Effects of Memantine
Source: Int J Mol Sci. 2025 Feb 14;26(4):1634. doi: 10.3390/ijms26041634 (PMC11855568; doi:10.3390/ijms26041634)
Supplement: Supplementary file 1 [file ijms-26-01634-s001.zip › Supplementary Tables S1-S18.pdf]

<u>Supplementary Tables S1–S18</u>

Table S1a. mRNA expression levels of CHRNA7 in cerebral grey matter (CGM), subcortical white matter (SCWM) and cerebellum (Ce) in four analyzed rat groups.

(all data were expressed as means ± standard deviation; *p*—level of statistical significance in different brain areas and animal groups)

Groups	CGM	SCWM	Ce	Statistics
young controls (young C)	3.0 ± 0.32	3.5 ± 0.08	2.8 ± 0.29	*p*=0.004*<br>*p*=0.003** (SCWM vs Ce)<br>*p*=0.017** (SCWM vs CMG)
old controls (old C)	2.8 ± 0.14	3.2 ± 0.07***(#'##)	2.7 ± 0.24	*p*=0.0007*<br>*p*=0.001** (SCWM vs Ce)<br>*p*=0.002** (SCWM vs CMG)
water group (water G)	3.0 ± 0.40	3.5 ± 0.16	2.9 ± 0.30	*p*=0.013*<br>*p*=0.02** (SCWM vs Ce)
memantine group (memantine G)	2.9 ± 0.15	3.5 ± 0.05	2.8 ± 0.32	*p*<0.0001*<br>*p*=0.0001** (SCWM vs Ce)<br>*p*=0.0005** (SCWM vs CMG)

p—level of statistical significance: * for ANOVA test - analyses differences in data between three brain regions (CGM, SCWM, Ce) within one of selected animal groups (young C, old C, water G or memantine G); ** for POST HOC TEST (Tukey test or Dunn test for, respectively, parametric or nonparametric data distributions); *** for Mann-Whitey test – analyses differences in data between two selected animal groups within each of three brain regions (CGM, SCWM, Ce); # *p*=0.008 (old C vs young C in SCWM); ## *p*=0.032 (old C vs water G in SCWM);

Table S1b. Protein expression levels of CHRNA7 in cerebral grey matter (CGM), subcortical white matter (SCWM) and cerebellum (Ce) in four analyzed rat groups.

(all data were expressed as means ± standard deviation, *p*—level of statistical significance in different brain areas and animal groups))

Groups	CGM	SCWM	Ce	*Statistics*
young controls (young C)	40.9 ± 4.31	35.0 ± 3.38	39.7 ± 4.37	NS*
old controls (old C)	35.5 ± 2.53	30.9 ± 1.50***#	31.3 ± 1.51***##	NS*
water group (water G)	47.9 ± 4.98	45.4 ± 2.85	45.3 ± 3.83	NS*
memantine group (memantine G)	43.8 ± 1.27	44.1 ± 1.17	43.2 ± 1.56	NS*

NS*—statistically insignificant difference in *ANOVA test - analyses differences in data between three brain regions (CGM, SCWM, Ce) within one of selected animal groups (young C, old C, water G or memantine G); *p*—level of statistical significance *** for Mann-Whitey test – analyses differences in data between two selected animal groups within each of three brain regions (CGM, SCWM, Ce); # *p*=0.008 (old C vs water G in SCWM); ## *p*=0.015 (old C vs water G in Ce);

Table S1c. Correlation between CHRNA7 mRNA and CHRNA7 protein in cerebral grey matter (CGM), subcortical white matter (SCWM) and cerebellum (Ce) in four analyzed rat groups

Groups		CGM	SCWM	Ce
young controls (young C)	R	0.999	0.100	0.999
	P	<0.0001	0.873	<0.0001
old controls (old C)	R	0.949	-0.100	0.821
	P	0.014	0.873	0.089
water group (water G)	R	0.999	0.700	0.999
	P	<0.0001	0.188	<0.0001
memantine group (memantine G)	R	0.600	0.406	0.943
	P	0.208	0.425	0.005

R—coefficient of Pearson or Spearman (for, respectively, parametric or nonparametric data distributions); *p*—level of statistical significance

Table S2a. mRNA expression levels of CHRNA4 in cerebral grey matter (CGM), subcortical white matter (SCWM) and cerebellum (Ce) in four analyzed rat groups.
(all data were expressed as means ± standard deviation in different brain areas and animal groups)

Groups	CGM	SCWM	Ce	*Statistics*
young controls (young C)	3.4 ± 0.20	3.4 ± 0.30	3.1 ± 0.13	NS*
old controls (old C)	3.2 ± 0.40	3.4 ± 0.14	3.1 ± 0.21	NS*
water group (water G)	3.5 ± 0.20	3.3 ± 0.48	3.0 ± 0.27	NS*
memantine group (memantine G)	3.7 ± 0.11	3.3 ± 0.36	3.3 ± 0.14	NS*

NS*—statistically insignificant difference in *ANOVA test - analyses differences in data between three brain regions (CGM, SCWM, Ce) within one of selected animal groups (young C, old C, water G or memantine G);

Table S2b. mRNA expression levels of CHRNB2 in cerebral grey matter (CGM), subcortical white matter (SCWM) and cerebellum (Ce) in four analyzed rat groups.
(all data were expressed as means ± standard deviation, *p*—level of statistical significance in different brain areas and animal groups)

Groups	CGM	SCWM	Ce	*Statistics*
young controls (young C)	2.4 ± 0.58	2.6 ± 0.96	2.1 ± 0.79	NS*
old controls (old C)	2.0 ±1.15	3.2 ± 1.05	3.1 ± 0.26	NS*
water group (water G)	2.8 ± 0.77	3.6 ± 1.47	2.8 ± 0.55	NS*
memantine group (memantine G)	3.3 ± 0.21	2.6 ± 1.26	2.3 ± 0.83	NS*

NS*—statistically insignificant difference in *ANOVA test - analyses differences in data between three brain regions (CGM, SCWM, Ce) within one of selected animal groups (young C, old C, water G or memantine G);

Table S3. Protein expression levels of TP53 in cerebral grey matter (CGM), subcortical white matter (SCWM) and cerebellum (Ce) in four analyzed rat groups.

(all data were expressed as means ± standard deviation, *p*—level of statistical significance in different brain areas and animal groups)

Groups	CGM	SCWM	Ce	*Statistics*
young controls (young C)	45.2 ± 4.11	57.8 ± 7.14	50.5 ± 7.61	NS*
old controls (old C)	34.3 ± 3.80	47.1 ± 3.31	47.4 ± 1.96	NS*
water group (water G)	42.3 ± 4.18	40.4 ± 3.74	47.2 ± 5.87	NS*
memantine group (memantine G)	46.7 ± 4.49	50.2 ± 5.51	61.2 ± 6.32	NS*

NS*—statistically insignificant difference in *ANOVA test - analyses differences in data between three brain regions (CGM, SCWM, Ce) within one of selected animal groups (young C, old C, water G or memantine G);

Table S4a. mRNA expression levels of OGG1 in cerebral grey matter (CGM), subcortical white matter (SCWM) and cerebellum (Ce) in four analyzed rat groups.

(all data were expressed as means ± standard deviation, *p*—level of statistical significance in different brain areas and animal groups)

Groups	CGM	SCWM	Ce	*Statistics*
young controls (young C)	3.0 ± 0.40***#	2.4 ± 0.23	2.3 ± 0.64	NS*
old controls (old C)	2.0 ± 0.63	2.6 ± 0.62	1.7 ± 0.43	NS*
water group (water G)	2.7 ± 0.39	2.7 ± 0.52	1.8 ± 0.54	NS*
memantine group (memantine G)	2.7 ± 0.31	2.2 ± 0.43	1.9 ± 0.70	NS*

NS*—statistically insignificant difference in *ANOVA test - analyses differences in data between three brain regions (CGM, SCWM, Ce) within one of selected animal groups (young C, old C, water G or memantine G); p—level of statistical significance *** for Mann-Whitey test – analyses differences in data between two selected animal groups within each of three brain regions (CGM, SCWM, Ce); # p=0.032 (old C vs young C in CGM)

Table S4b. Protein expression levels of OGG1 in cerebral grey matter (CGM), subcortical white matter (SCWM) and cerebellum (Ce) in four analyzed rat groups.
(all data were expressed as means ± standard deviation, *p*—level of statistical significance in different brain areas and animal groups)

Groups	CGM	SCWM	Ce	*Statistics*
young controls (young C)	42.2 ± 5.32	37.1 ± 3.53	36.4 ± 2.59	NS*
old controls (old C)	29.1 ± 1.27***#	32.2 ± 1.25	34.1 ± 3.80	NS*
water group (water G)	41.6 ± 6.88	30.1 ± 4.55	34.2 ± 3.40	NS*
memantine group (memantine G)	37.9 ± 4.21	30.8 ± 2.98	33.5 ± 2.51	NS*

NS*—statistically insignificant difference in *ANOVA test - analyses differences in data between three brain regions (CGM, SCWM, Ce) within one of selected animal groups (young C, old C, water G or memantine G); *p*—level of statistical significance *** for Mann-Whitney test – analyses differences in data between two selected animal groups within each of three brain regions (CGM, SCWM, Ce); # *p*=0.037 (old C vs young C in CGM);

Table S5. Protein expression levels of TNFα in cerebral grey matter (CGM), subcortical white matter (SCWM) and cerebellum (Ce) in four analyzed rat groups.
(all data were expressed as means ± standard deviation, *p*—level of statistical significance in different brain areas and animal groups)

Groups	CGM	SCWM	Ce	*Statistics*
young controls (young C)	30.7 ± 0.71	38.3 ± 2.22	40.9 ± 3.58	*p*=0.03*<br>*p*=0.03** (CGM vs Ce)
old controls (old C)	44.6 ± 5.37***#	36.3 ± 3.77	39.3 ± 1.65	NS*
water group (water G)	31.2 ± 3.16	31.6 ± 1.92	35.1 ± 1.45	NS*
memantine group (memantine G)	36.0 ± 1.12	32.5 ± 2.58	40.7 ± 4.60	NS*

p—level of statistical significance: * for ANOVA test - analyses differences in data between three brain regions (CGM, SCWM, Ce) within one of selected animal groups (young C, old C, water G or memantine G); ** for POST HOC TEST (Tukey test or Dunn test for, respectively, parametric or nonparametric data distributions); *** for Mann-Whitey test – analyses differences in data between two selected animal groups within each of three brain regions (CGM, SCWM, Ce); # *p*=0.008 (old C vs young C in CGM); NS*—statistically insignificant difference in ANOVA test

Table S6. Levels of 8-oxo-2′dG in cerebral grey matter (CGM), subcortical white matter (SCWM) and cerebellum (Ce) in four analyzed rat groups.
(the results represent ratios of 8-oxo2′dG/dG (×10^{-4}), all data were expressed as means ± standard deviation, *p*—level of statistical significance in different brain areas and animal groups)

Groups	CGM	SCWM	Ce	*Statistics*
young controls (young C)	36.8 ± 15.86	58.7 ± 21.33	8.9 ± 1.50	*p*=0.004*<br>*p*=0.03** (CGM vs Ce)<br>*p*=0.0007** (SCWM vs Ce)
old controls (old C)	44.7 ± 6.89	61.8 ± 43.65	13.5 ± 9.62	*p*=0.002*<br>*p*=0.03** (SCWM vs Ce)
water group (water G)	122.9 ± 130.25	91.6 ± 53.29	58.3 ± 50.39	NS*
memantine group (memantine G)	24.0 ± 12.17	22.2 ± 8.12	22.2 ± 8.12***#	NS*

p—level of statistical significance: * for ANOVA test - analyses differences in data between three brain regions (CGM, SCWM, Ce) within one of selected animal groups (young C, old C, water G or memantine G); ** for POST HOC TEST (Tukey test or Dunn test for, respectively, parametric or nonparametric data distributions); *** for Mann-Whitey test – analyses differences in data between two selected animal groups within each of three brain regions (CGM, SCWM, Ce); # *p*=0.009 (memantine G vs water G in Ce); NS—statistically insignificant difference in *ANOVA test

Table S7. Correlation between TP53 and TNF*α* proteins in cerebral grey matter (CGM), subcortical white matter (SCWM) and cerebellum (Ce) in four analyzed rat groups.
(the Spearman rank (R Spearman) correlation test)

Groups		CGM	SCWM	Ce
young controls (young C)	R	0.400	0.980	0.811
	P	0.504	0.003	0.096
old controls (old C)	R	0.070	0.204	-0.141
	P	0.911	0.740	0.821
water group (water G)	R	0.325	0.875	-0.352
	P	0.593	0.052	0.561
memantine group (memantine G)	R	-0.487	-0.565	-0.491
	P	0.328	0.242	0.323

R—coefficient of Pearson or Spearman (for, respectively, parametric or nonparametric data distributions); *p*—level of statistical significance

Table S8. Correlation between OGG1 and TP53 proteins in cerebral grey matter (CGM), subcortical white matter (SCWM) and cerebellum (Ce) in four analyzed rat groups

Groups		CGM	SCWM	Ce
young controls (young C)	R	0.189	-0.405	0.515
	P	0.761	0.499	0.374
old controls (old C)	R	-0.506	0.854	-0.746
	P	0.385	0.065	0.148
water group (water G)	R	-0.336	-0.043	-0.914
	P	0.580	0.945	0.030
memantine group (memantine G)	R	-0.118	0.522	-0.554
	P	0.823	0.288	0.254

R—coefficient of Pearson or Spearman (for, respectively, parametric or nonparametric data distributions); *p*—level of statistical significance

Table S9. Correlation between 8-oxo-2’dG and OGG1 protein in cerebral grey matter (CGM), subcortical white matter (SCWM) and cerebellum (Ce) in four analyzed rat groups
(the Spearman rank (R Spearman) correlation test)

Groups		CGM	SCWM	Ce
young controls (young C)	R	0.700	0.000	-0.300
	P	0.188	1.000	0.624
old controls (old C)	R	0.400	-0.500	0.844
	P	0.505	0.391	0.072
water group (water G)	R	0.500	-0.100	-0.308
	P	0.391	0.873	0.614
memantine group (memantine G)	R	0.257	0.086	0.257
	P	0.623	0.872	0.623

R—coefficient of Pearson or Spearman (for, respectively, parametric or nonparametric data distributions); *p*—level of statistical significance

Table S10. Correlation between 8-oxo-2′dG and TNFα protein in cerebral grey matter (CGM), subcortical white matter (SCWM) and cerebellum (Ce) in four analyzed rat groups (the Spearman rank (R Spearman) correlation test)

Groups		CGM	SCWM	Ce
young controls (young C)	R	0.700	0.600	-0.100
	P	0.188	0.285	0.873
old controls (old C)	R	-0.800	0.700	0.788
	P	0.104	0.188	0.017
water group (water G)	R	-0.500	0.000	0.300
	P	0.391	1.000	0.624
memantine group (memantine G)	R	0.371	-0.891	0.714
	P	0.468	0.017	0.111

R—coefficient of Pearson or Spearman (for, respectively, parametric or nonparametric data distributions); *p*—level of statistical significance

Table S11. Correlation between OGG1 mRNA and TNFα protein in cerebral grey matter (CGM), subcortical white matter (SCWM) and cerebellum (Ce) in four analyzed rat groups (the Spearman rank (R Spearman) correlation test)

Groups		CGM	SCWM	Ce
young controls (young C)	R	0.900	-0.200	0.500
	P	0.037	0.747	0.391
old controls (old C)	R	-0.900	-0.800	0.800
	P	0.037	0.104	0.104
water group (water G)	R	-0.600	-0.800	0.700
	P	0.285	0.104	0.188
memantine group (memantine G)	R	0.429	-0.086	0.314
	P	0.397	0.872	0.544

R—coefficient of Pearson or Spearman (for, respectively, parametric or nonparametric data distributions); *p*—level of statistical significance

Table S12. Correlation between TP53 protein and CHRNA7 protein in cerebral grey matter (CGM), subcortical white matter (SCWM) and cerebellum (Ce) in four analyzed rat groups
(the Spearman rank (R Spearman) correlation test)

Groups		CGM	SCWM	Ce
young controls (young C)	R	0.917	-0.800	0.300
	P	0.028	0.104	0.624
old controls (old C)	R	0.100	0.300	0.975
	P	0.873	0.624	0.005
water group (water G)	R	0.500	-0.100	0.500
	P	0.391	0.873	0.391
memantine group (memantine G)	R	-0.200	0.257	-0.657
	P	0.704	0.623	0.156

R—coefficient of Pearson or Spearman (for, respectively, parametric or nonparametric data distributions); *p*—level of statistical significance

Table S13. Correlation between CHRNA7 mRNA and OGG1 mRNA in cerebral grey matter (CGM), subcortical white matter (SCWM) and cerebellum (Ce) in four analyzed rat groups
(the Spearman rank (R Spearman) correlation test)

Groups		CGM	SCWM	Ce
young controls (young C)	R	0.600	-0.897	0.000
	P	0.285	0.039	1.000
old controls (old C)	R	0.316	-0.949	0.000
	P	0.604	0.014	1.000
water group (water G)	R	0.885	0.300	0.100
	P	0.046	0.624	0.873
memantine group (memantine G)	R	-0.200	0.319	-0.200
	P	0.704	0.538	0.704

R—coefficient of Pearson or Spearman (for, respectively, parametric or nonparametric data distributions); *p*—level of statistical significance

Table S14. Correlation between CHRNA4 mRNA and OGG1 mRNA in cerebral grey matter (CGM), subcortical white matter (SCWM) and cerebellum (Ce) in four analyzed rat groups (the Spearman rank (R Spearman) correlation test)

Groups		CGM	SCWM	Ce
young controls (young C)	R	0.300	0.200	0.961
	P	0.624	0.747	0.0009
old controls (old C)	R	0.884	0.300	0.000
	P	0.046	0.624	1.000
water group (water G)	R	-0.300	0.900	0.300
	P	0.624	0.037	0.624
memantine group (memantine G)	R	0.371	0.257	0.429
	P	0.468	0.623	0.397

R—coefficient of Pearson or Spearman (for, respectively, parametric or nonparametric data distributions); *p*—level of statistical significance

Table S15. Correlation between CHRNB2 mRNA and OGG1 mRNA in cerebral grey matter (CGM), subcortical white matter (SCWM) and cerebellum (Ce) in four analyzed rat groups (the Spearman rank (R Spearman) correlation test)

Groups		CGM	SCWM	Ce
young controls (young C)	R	0.100	0.100	-0.900
	P	0.873	0.873	0.037
old controls (old C)	R	0.700	0.100	0.200
	P	0.188	0.873	0.747
water group (water G)	R	-0.100	0.300	0.100
	P	0.873	0.624	0.873
memantine group (memantine G)	R	0.143	0.300	0.314
	P	0.787	0.624	0.544

R—coefficient of Pearson or Spearman (for, respectively, parametric or nonparametric data distributions); *p*—level of statistical significance

Table S16. Correlation between CHRNA7 protein and 8-oxo-2′dG in cerebral grey matter (CGM), subcortical white matter (SCWM) and cerebellum (Ce) in four analyzed rat groups (the Spearman rank (R Spearman) correlation test)

Groups		CGM	SCWM	Ce
young controls (young C)	R	-0.700	-0.887	-0.700
	P	0.188	0.045	0.188
old controls (old C)	R	0.600	-0.500	-0.205
	P	0.285	0.391	0.741
water group (water G)	R	0.200	-0.500	0.400
	P	0.747	0.391	0.505
memantine group (memantine G)	R	-0.143	-0.371	0.600
	P	0.787	0.468	0.208

R—coefficient of Pearson or Spearman (for, respectively, parametric or nonparametric data distributions); *p*—level of statistical significance

Table S17. Correlation between CHRNA4 mRNA and 8-oxo-2′dG in cerebral grey matter (CGM), subcortical white matter (SCWM) and cerebellum (Ce) in four analyzed rat groups (the Spearman rank (R Spearman) correlation test)

Groups		CGM	SCWM	Ce
young controls (young C)	R	0.100	-0.600	0.100
	P	0.873	0.285	0.873
old controls (old C)	R	0.800	0.300	0.300
	P	0.104	0.624	0.624
water group (water G)	R	-0.500	-0.300	-0.300
	P	0.391	0.624	0.624
memantine group (memantine G)	R	-0.086	-0.829	-0.657
	P	0.328	0.042	0.156

R—coefficient of Pearson or Spearman (for, respectively, parametric or nonparametric data distributions); *p*—level of statistical significance

Table S18. Correlation between CHRNB2 mRNA and 8-oxo-2′dG in cerebral grey matter (CGM), subcortical white matter (SCWM) and cerebellum (Ce) in four analyzed rat groups (the Spearman rank (R Spearman) correlation test)

Groups		CGM	SCWM	Ce
young controls (young C)	R	-0.300	-0.800	-0.300
	P	0.624	0.104	0.624
old controls (old C)	R	0.600	0.100	-0.300
	P	0.285	0.873	0.624
water group (water G)	R	-0.946	-0.100	-0.600
	P	0.015	0.873	0.285
memantine group (memantine G)	R	0.784	-0.314	0.143
	P	0.065	0.544	0.787

R—coefficient of Pearson or Spearman (for, respectively, parametric or nonparametric data distributions); *p*—level of statistical significance
